# Development of manganese ferrite coated with Prussian blue as an efficient contrast agent for applications in magnetic resonance imaging

**DOI:** 10.1038/s41598-025-98348-7

**Published:** 2025-04-23

**Authors:** Ágnes Maria Ilosvai, Fatemeh Heydari, László Forgách, Noémi Kovács, Krisztián Szigeti, Domokos Máthé, Ferenc Kristály, Lajos Daróczi, Béla Viskolcz, Miklós Nagy, Miklós Németh, Tamás Ollár, László Vanyorek

**Affiliations:** 1 Institute of Chemistry, University of Miskolc, Miskolc-Egyetemváros, Miskolc, 3515 Hungary; 2 Higher Education and Industrial Cooperation Centre, University of Miskolc, Miskolc, 3515 Hungary; 3 Department of Biophysics and Radiation Biology, Semmelweis University, Budapest, 1094 Hungary; 4 In Vivo Imaging Advanced Core Facility, Hungarian Center of Excellence for Molecular Medicine (HCEMM), Szeged, 6728 Hungary; 5 Institute of Mineralogy and Geology, University of Miskolc, Miskolc, 3515 Hungary; 6 Department of Solid State Physics, University of Debrecen, P.O. Box 2, Debrecen, 4010 Hungary; 7 Surface Chemistry and Catalysis Department, Institute for Energy Security and Environmental Safety, HUN-REN Centre for Energy Research, Budapest, 1121 Hungary

**Keywords:** Magnetic nanoparticles, Prussian blue, MRI, Ferrite, Non-toxic, Medical research, Materials science

## Abstract

Magnetic iron oxide nanoparticles are frequently utilized as contrast agents in magnetic resonance imaging (MRI). However, the release of iron ions leads to the formation of reactive oxygen species (ROS), resulting in cell damage. In this study, we developed MRI contrast agents containing amine-functionalized MnFe_2_O_4_ nanoparticles with a Prussian blue (PB) surface coating to enhance their biocompatibility. The prepared MNPs were embedded in polyvinylpyrrolidone and dried. The resulting crystals can be redispersed in water immediately before use, forming a stable colloid. The particle size of nanoparticles (43 ± 13 nm) is suitable for the intended application. The values of Hc (52 Oe) and Mr (3.7 emu/g) for the particles indicate a soft ferromagnetic nature. The coating of the particles with PB results in a significant reduction of their toxicity, as evidenced by a toxicological test on HEK293 cells. This colloid was tested in vitro as an MRI contrast agent as well as in healthy animal. The longitudinal relaxivity (r_1_) of the PB-MnFe_2_O_4_-NH_2_ sample was determined to be 0.01 (mg/mL)^−1^ms^− 1^. The transversal relaxivity was measured as well (r_2_: 0.77 (mg/mL)^−1^ms^− 1^ and r_2_*: 1.48 (mg/mL)^−1^ms^− 1^, which were in the same range as Feraheme and Endorem. Prussian blue-coated MnFe_2_O_4_ emerges as a promising T2-weighted contrast material, representing a novel combination of two well-known contrast-capable materials.

## Introduction

Various magnetic nanoparticles are being investigated in many biomedical fields, such as MRI contrast agents and targeted drug delivery systems, owing to their biocompatibility and potential for the controlled release of therapeutic agents and hyperthermia treatment^1–6^. Magnetic nanomaterials are used in various sample preparation operations in which the component to be measured is extracted by magnetic separation, such as the isolation of macromolecules, for example, DNA and glycans^7–10^. In magnetic resonance imaging, Gd chelate complexes are increasingly being displaced by iron oxide-based magnetic nanoparticles in clinical applications. The use of Gd-based contrast agents raises toxicity issues due to the unexpected release of free Gd ions in the body^11,12^. The iron oxide-based magnetic nanoparticles, especially superparamagnetic ones, including magnetite, maghemite, and the various ferrite nanoparticles, are well usable as MRI contrast agent instead the above-mentioned Gd complex, owing to their biocompatibility^13,14^. However, problems can also arise with magnetic iron oxide contrast agents. Undesirable side effects have been reported with commonly used commercially available iron oxide contrast agents, including local pain, hypotension, hypersensitivity, anaphylactic shock, vasodilatation, and paresthesia^14^. Due to the health risks and side effects mentioned above, ferumoxide (Feridex, Endorem) and ferucarbotran (Ciavist, Resovist) were withdrawn from the market in 2008 and 2009^14–16^. For magnetite and maghemite-based contrast agents, the aim should be to ensure successful imaging in MRI scans with the lowest possible dose. These contrast agents can cause iron overload, resulting in the increased production of reactive oxygen species (ROS) via Fenton and Haber–Weiss reactions, which can cause intracellular damage^17–24^. The toxicity of magnetic nanoparticles can be reduced by adding biocompatible layers on the surface (e.g., HSA)^25,26^. Another way to reduce toxicity is to use complexing agents to make the surface of the particles chemically resistant, preventing iron leaching. A promising complexing agent is potassium hexacyanoferrate, which forms a chemically inert complex with Fe(II) and other divalent transition metal ions. Prussian blue, also known as iron(II) iron(III) octadecacyanide (Fe^III^_4_[Fe^II^(CN)_6_]_3_), is a biocompatible complex with a wide range of applications in both nanomedicine and imaging diagnostics^27^. Maghemite nanoparticles treated with a potassium hexacyanoferrate complexing agent can be coated with a Prussian blue layer on their surface, thus biocompatibility is ensured^28^. Prussian blue analogues are easy to prepare since potassium hexacyanoferrate complexes efficiently with other divalent transition metal ions such as zinc(II), copper(II), manganese(II), cobalt(II), etc., forming chemically stable transitional metal hexacyanoferrate complexes^29–31^. The production of the Pussian blue coating mentioned for maghemite may be feasible on other magnetic nanoparticles such as transition metal ferrites (MFe_2_O_4_, where M: Cu(II), Mn(II), Co(II), Zn(II), etc.), which are spinel-type magnetic metal oxides^32–34^. These magnetic nanomaterials are well-suited as T2 MRI contrast agents^35–37^. The aforementioned MFe_2_O_4_ spinel-type magnetic nanoparticles can be prepared in various ways, including solvothermal, coprecipitation, sonochemical, microemulsion, or hydrothermal synthesis^38–43^. Amine functional groups^38^ or various coatings^39^ can be formed on the surface of ferrite nanoparticles. An efficient and high-yield synthesis method for amine-functionalized magnesium ferrite nanoparticles is the solvothermal method^44^. In the solvothermal method, ethylene glycol (EG), polyethylene glycol (PEG), or a mixture of ethanol and EG-water in the presence of surfactants and sodium acetate are used as the reaction medium. These synthesis methods yield nanoparticles with similar morphologies^45–47^.

Our work aims to develop a Prussian blue-coated MnFe_2_O_4_ type T2 contrast material embedded in a biocompatible polymer, which can be stored in solid form for a long time (for years) and easily redissolved in an aqueous medium. For nanoparticles embedded and dried in a PVP polymer, a shelf life of several years can be designated. However, when SPIO colloidal systems are in an aqueous environment, the potential for aggregation must be considered. This issue is not present in dried and re-dispersible contrast agents. In the case of solid, well dispersible formulations, concerns regarding the adverse effects of temperature fluctuations (or freezing) on colloid stability are not relevant, in contrast to the case of aqueous medium contrast agents.

## Results and discussion

### Formation mechanism of the amine functionalized manganese ferrite nanoparticles

The formation of manganese ferrite nanoparticles is assisted by the transformation of monoethanolamine (MEA), as the presence of manganese(II) ions reduces the thermal decomposition temperature of MEA, releasing ammonia^48^. Furthermore, Rochell has shown that the presence of Fe(III) ions promotes the oxidative degradation of MEA. During this process, an aminium radical is formed, which is deprotonated to an imine radical that is further degraded to ammonia and aldehyde (hydroxyacetaldehyde) via imine^49^. The alkaline conditions lead to the formation of Fe(OH)_3_ and Mn(OH)_2_, which can be transformed into MnFe_2_O_4_ through dehydration.

$$\:{\text{N}\text{H}}_{2\:}{\text{C}\text{H}}_{2}{\text{C}\text{H}}_{2}\text{O}\text{H}\underrightarrow{{\text{F}\text{e}}^{3+}{and\:-H}^{+}}\:{\text{H}}_{2}{\text{N}}^{*}{=\text{C}\text{H}-\text{C}\text{H}}_{2}-\text{O}\text{H}+\:{\text{H}}_{2}\text{O}\:\underrightarrow{{\text{F}\text{e}}^{3+}{and\:-H}^{+}}\:\text{H}\text{O}{\text{C}\text{H}}_{2}\text{C}\text{H}\text{O}+{\:\text{N}\text{H}}_{3}$$

$$\:{\text{N}\text{H}}_{3}+\:{\text{H}}_{2}\text{O}\:\to\:\:{\text{N}\text{H}}_{4}^{+}+\:{\text{O}\text{H}}^{-}$$

$$\:{\text{F}\text{e}}^{3+}+\:3\:{\text{O}\text{H}}^{-}\:\to\:\:{\text{F}\text{e}\left(\text{O}\text{H}\right)}_{3}$$

$$\:{\text{M}\text{n}}^{2+}+\:{2\:\text{O}\text{H}}^{-}\:\to\:\:{\text{M}\text{n}\left(\text{O}\text{H}\right)}_{2}$$

$$\:{\text{M}\text{n}\left(\text{O}\text{H}\right)}_{2}+\:{2\:\text{F}\text{e}\left(\text{O}\text{H}\right)}_{3}\:\to\:\:{\text{M}\text{n}\text{F}\text{e}}_{2}{\text{O}}_{4}+\:{4\:\text{H}}_{2}\text{O}$$

### Characterization of the MnFe_2_O_4_-NH_2_ nanoparticles

**Fig. 1:**
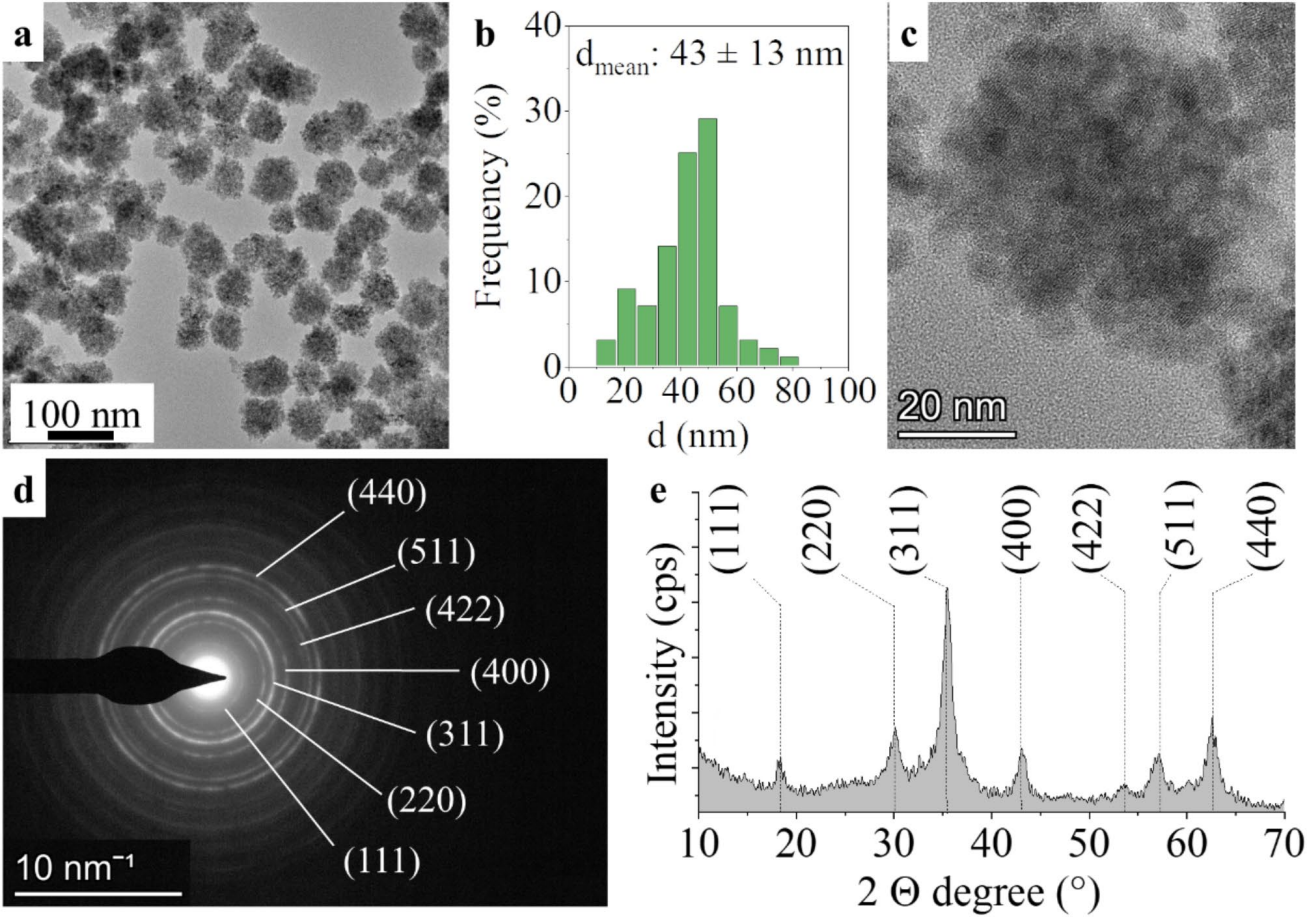
TEM picture of the MnFe_2_O_4_-NH_2_ clustering structures (**a**) and their size distribution histogram (**b**) HRTEM picture of the cluster which built from smaller, individual manganese ferrite nanoparticles (**c**) SAED picture of the nanoparticles with the Miller indices (**d**) and the Miller indexed reflexions on X-ray diffractogram (**e**) of the MnFe_2_O_4_-NH_2_ nanoparticles.

TEM measurements were carried out on manganese ferrite nanoparticles, which formed clustering structures with a diameter of 43 ± 13 nm (Fig. 1a and b). These clustering structures show high integrity, with spherical structures built from smaller individual ferrite nanoparticles (Fig. 1c). The production of the same aggregated spherical particles has been reported by several research groups in the polyol-based solvothermal synthesis of manganese ferrite^50–52^. The crystallization of the smaller ferrite nanoparticles into the greater spherical structures seen in the TEM image is a typical phenomenon for other ferrites also (NiFe_2_O_4_, CoFe_2_O_4_, MgFe_2_O_4_, ZnFe_2_O_4_ etc.) and magnetite prepared by polyol method in ethylene glycol medium. Nonkumwong et al. have prepared amine-functionalized magnesium ferrite nanoparticles using the same method as ours, with morphologies very similar to those of our manganese ferrite nanoparticles^53^. Similar spherical aggregates were formed when urea (as an ammonia source) was used in the solvothermal synthesis of zinc ferrite, nickel ferrite and cobalt ferrite in ethylene glycol medium^54^. Similar spherical aggregates were also produced from copper ferrite by Castilla, using ethylene glycol sodium acetate and polyethylene glycol as reactants in addition to metal precursors^55^.

Based on the XRD results, the crystallite sizes were calculated using full width at half maximum (FWHM), with an average crystallite size of 5 ± 2 nm. The ferrite particles were examined by selected area electron diffraction (SAED), and the nanoparticles in the HRTEM pictures were identified as MnFe_2_O_4_ spinel (Fig. 1d). The measured d-spacing values were correlated with the d-values in X-ray databases, specifically the powder diffraction file of the jacobsite (PDF 74-2403) spinel structure (Fig. 1d). On the X-ray diffractogram, reflection peaks were located at 18.2° (111), 29.9° (220), 35.2° (311), 42.4° (400), 52.7° (422), 56.4° (511), and 61.6° (440) two theta degrees, which match the peaks corresponding to the manganese ferrite phase (PDF 74–2403) (Fig. 1e). No other metal oxide phases were found in the sample. The polyol-based solvothermal synthesis method is well suited for the production of pure ferrite phases, and there are many examples in the literature not only for manganese ferrite, but also for zinc ferrite, cobalt ferrite, nickel ferrite and magnesium ferrite, which can be produced without impurities^50,53,54^.

**Fig. 2:**
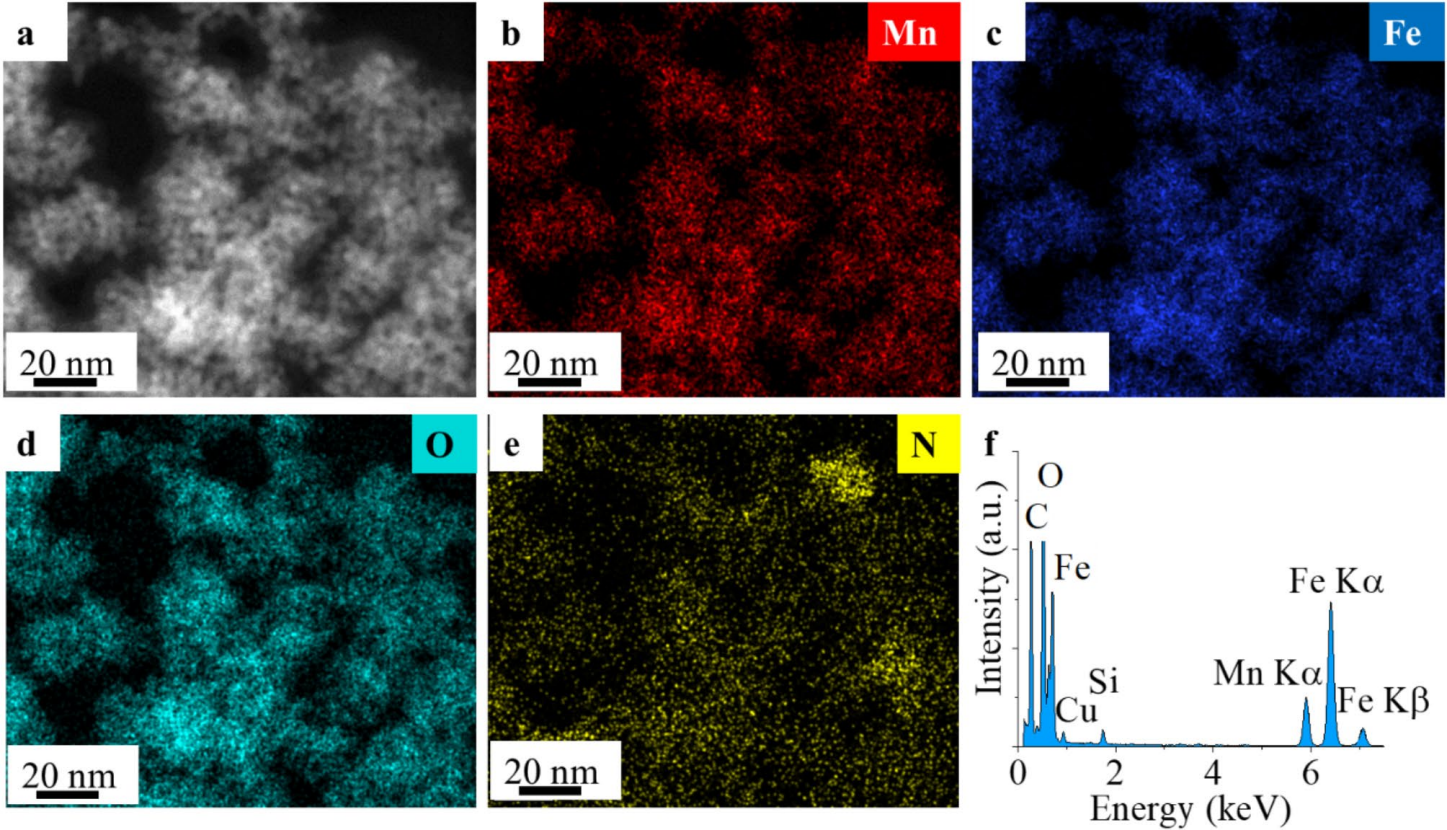
HAADF picture of the amine-functionalized ferrite particles (**a**) and their constituent elements (Mn, Fe, O, N) on element maps (**b**–**e**) and EDS with the components (**f**) of the MnFe_2_O_4_-NH_2_ nanoparticles.

In the high-angle annular dark-field (HAADF) image, the manganese ferrite nanoparticles appear in sharp contrast (Fig. 2a). The elemental maps show that the positions of iron, manganese, and oxygen elements are the same in the detected area (Fig. 2b, c and d). This suggests that iron, manganese, and oxygen are present as ferrite in the sample, with no signs of iron and manganese separately on the elemental map. The presence of nitrogen in the elemental maps can be explained by the presence of ethanolamine molecules on the surface of the MnFe_2_O_4_ nanoparticles (Fig. 2e). The energy-dispersive X-ray spectrum (EDS) identified the elements that make up the ferrite structure, namely manganese, iron, and oxygen (Fig. 2f). In addition to the ferrite components, a band of copper is also visible in the spectrum, due to the material of the sample holder grid used in electron microscopy. The source of the carbon is partly a carbon layer on the surface of the TEM grid and partly organic molecules (ethylene glycol and ethanolamine) adsorbed on the surface of the nanoparticles.

### Characterization of the Prussian blue modified MnFe_2_O_4_-NH_2_ nanoparticles

**Fig. 3:**
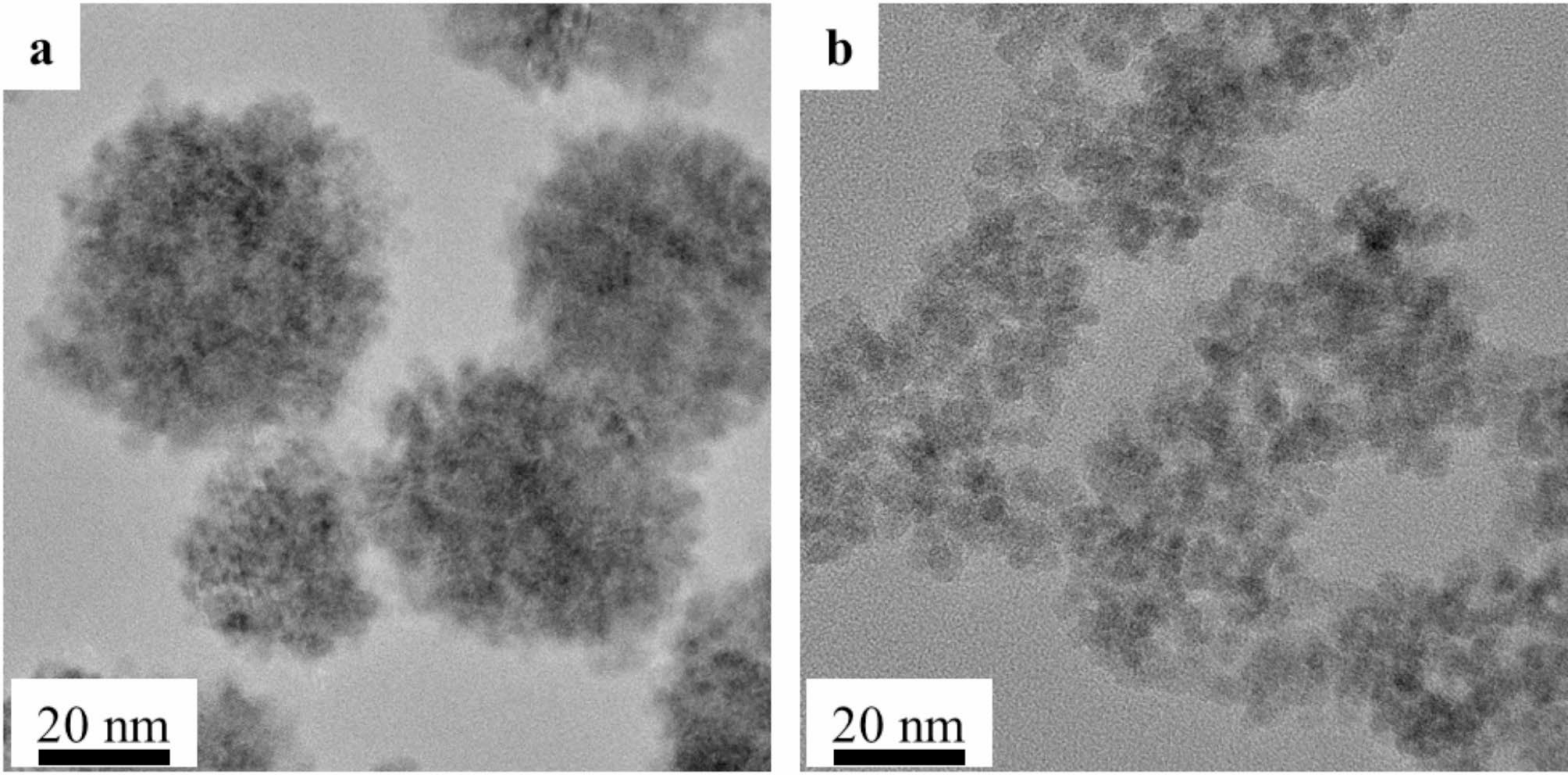
HRTEM picture of the untreated cluster structures MnFe_2_O_4_-NH_2_ particles, (**a**) and the PB-MnFe_2_O_4_-NH_2_ (**b**) nanoparticles, which have not maintained their integrity and have broken up into smaller nanoparticles.

The MnFe_2_O_4_-NH_2_ PB samples were also examined by HRTEM. The TEM images show that the morphological characteristics of the untreated and Prussian blue-coated manganese ferrite samples were different (Fig. 3a). As detailed above, the manganese ferrite nanoparticles, which are 5 ± 2 nm in size (as measured by XRD), crystallize into spherical aggregates that can be disintegrated by the complexing agent. The degree of integrity of the spherical nanoclusters was reduced in the case of the Prussian blue-coated sample (PB-MnFe_2_O_4_-NH_2_) (Fig. 3b). The nanoclusters seen in the untreated manganese ferrite (MnFe_2_O_4_-NH_2_) samples were dispersed into small crystallites by the complexation effect.

**Fig. 4:**
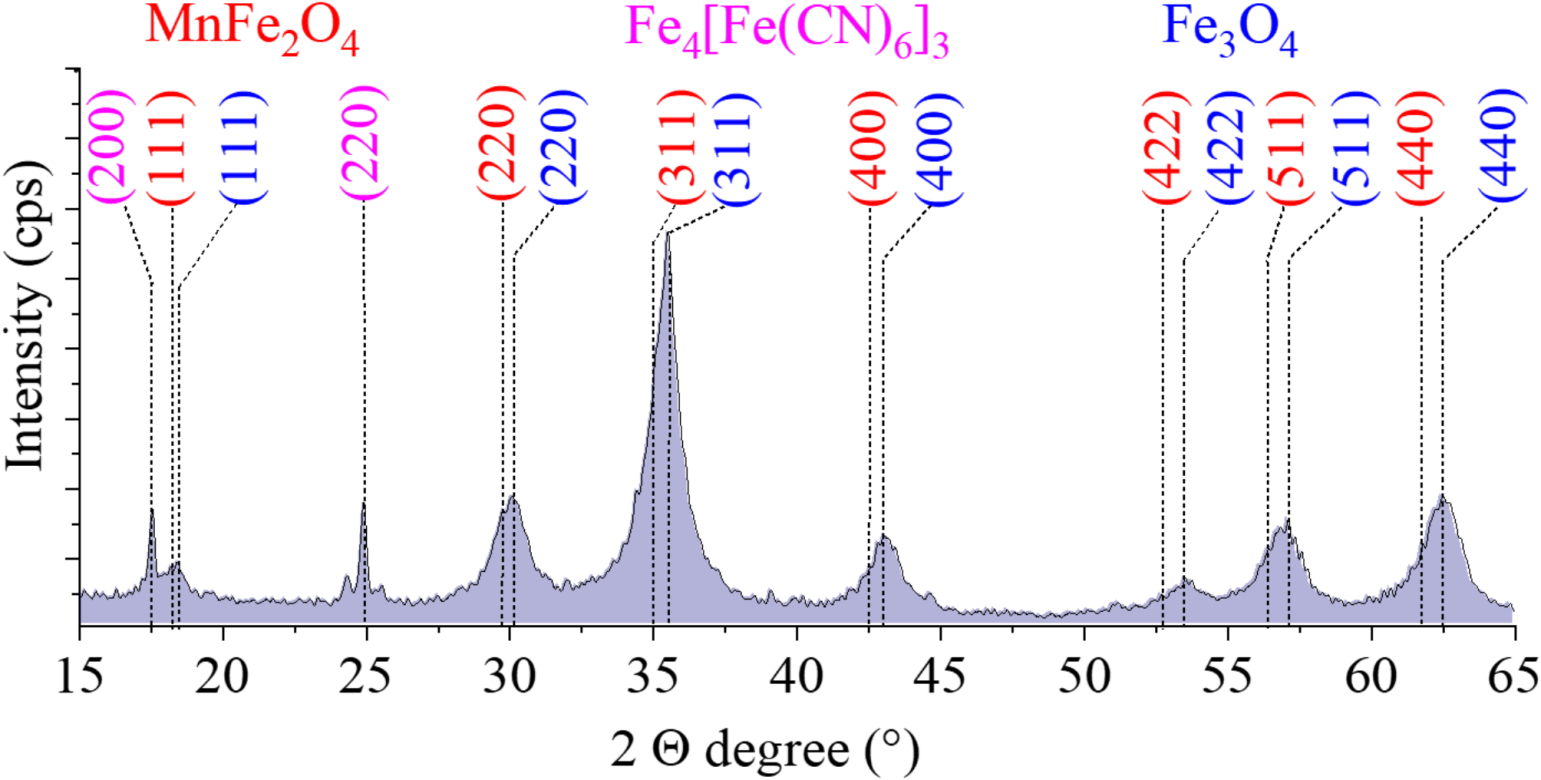
X-ray diffractogram of the PB-MnFe_2_O_4_-NH_2_ sample, and the Miller indexed reflexions of the crystal phases present in the sample such as magnetite, manganese ferrite and Prussian blue.

The phase structure of the PB-MnFe_2_O_4_-NH_2_ nanoparticles were examined by XRD (Fig. 4). The diffractogram identified reflections characteristic of the jacobsite (MnFe_2_O_4_) phase, located at 18.2° (111), 29.8° (220), 34.9° (311), 42.8° (400), 52.8° (422), 56.4° (511), and 61.7° (440) two theta degrees. The quantity of manganese ferrite is 43 wt% (PDF 74–2403) (Fig. 4). Other peaks are located at 17.6° (200) and 24.9° (220) two theta degrees, which belong to the Prussian blue, with a quantity of 7.3 wt% (PDF 73–0687). Gögen et al. observed also these reflexions in the case of Prussian blue-coated magnetite nanoparticles, at 17.40° and 24.76° (2Ɵ degrees)^56^.

However, owing to the deposition of Prussian blue on the manganese ferrite particles, the phase composition changed, forming other compounds alongside the Fe_4_[Fe(CN)_6_]_3_ phase. The characteristic reflections of magnetite were identified on the diffractogram at 18.5° (111), 30.1° (220), 35.5° (311), 43.1° (400), 53.4° (422), 57.1° (511), and 62.5° (440) two theta degrees (PDF 19–629). The quantity of magnetite is 45.4 wt%. Similar to our method, Thammawong et al. deposited a Prussian blue coating on the surface of magnetite nanoparticles using K_4_[Fe(CN)_6_] by ultrasonication^57^. They also examined the sample by XRD and found no impurities next to the Prussian blue and magnetite phases. The procedure we used differs in that after forming the Prussian blue coating on the surface of the MnFe_2_O_4_ particles by ultrasonication, we added FeCl_3_ solution to further increase the amount of Prussian blue on the nanoparticles. It can be assumed that a small fraction of the manganese ions was complexed with potassium hexacyanoferrate, so that part of the manganese ferrite was converted to magnetite, but manganese(II) ferrocyanide or other Prussian blue analogue of manganese was not detected in the XRD measurements. The absence of the Mn-containing Prussian blue complex is explained by the position of the Mn(II) ion in the Irving-Williams Series^58^. As the ionic radius decreases from Mn(II) to Zn(II) within the period, the stability of the complex increases. Furthermore, the crystal field stabilization energy (CFSE) increases from Mn(II) towards Ni(II), which also increases the stability of the complex. A similar observation was made by Risset et al. who synthesized Mn-containing Prussian blue analogue, Rb_1.6_Mn_4_[Fe(CN)_6_]_3.2_-4.8H_2_O particles, during the production of Rb_0.4_M_4_[Fe(CN)_6_]_2.8_·7.2H_2_O PBA hollow nanoparticles^59^. But manganese contained impurities were found in the sample at 4.3 wt% (potassium manganese chloride and manganese chloride urea complex) alongside the main phases (PDF 76–0970 and PDF 30–0810).

**Fig. 5:**
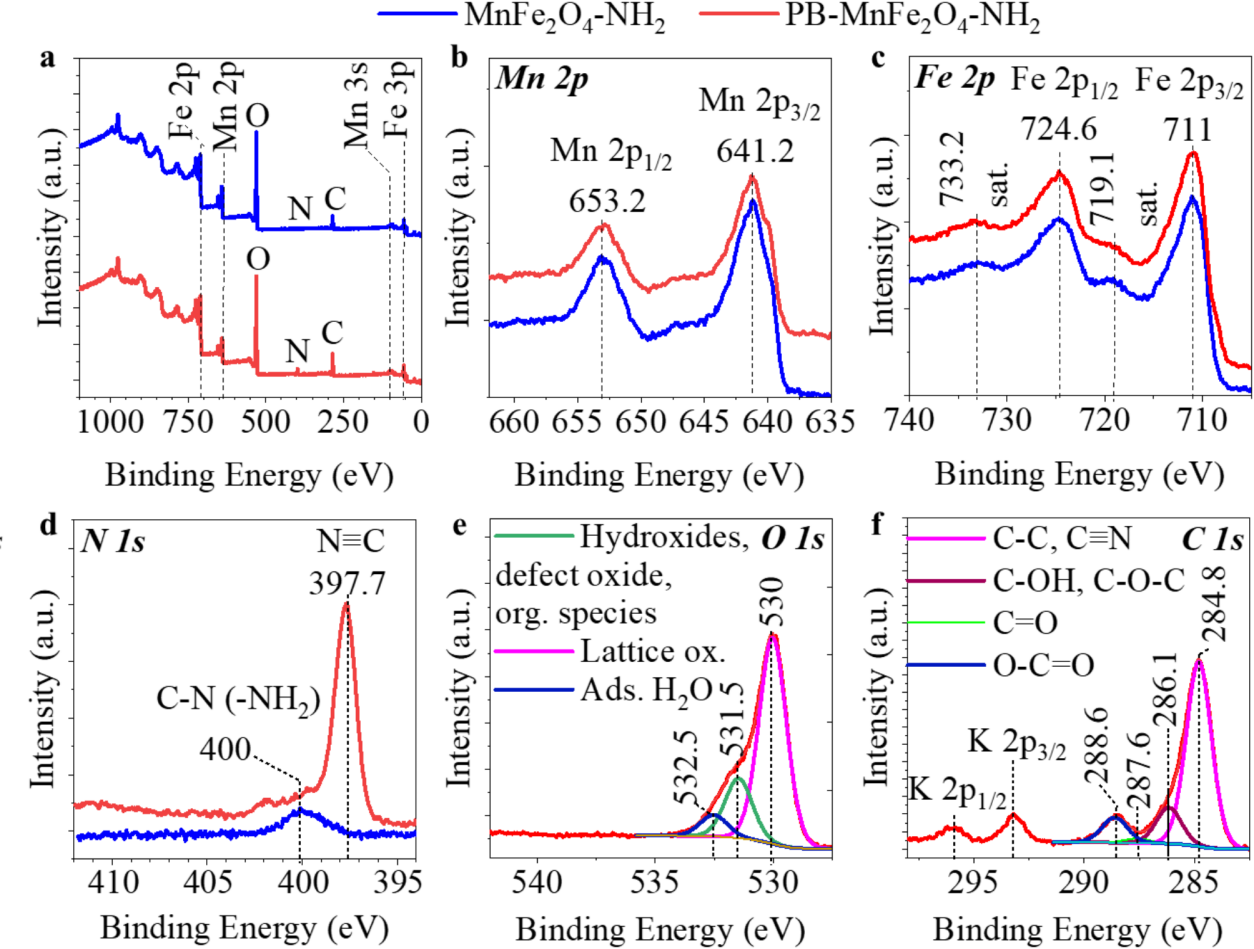
XPS results: survey spectrums of the MnFe_2_O_4_ and PB-MnFe_2_O_4_-NH_2_ nanoparticles (**a**) Mn 2p bands of the divalent Mn ions (Mn^2+^) in the MnFe_2_O_4_ spinel (**b**), Fe 2p peaks indicate the form of trivalent iron (Fe^3+^) (**c**), N 1s band origin from the amine functional groups (C-N) and N ≡ C bonds in the Fe^III^_4_[Fe^II^(CN)_6_]_3_ (**d**), O1 s band of the lattice oxide and functional groups in the PB-MnFe_2_O_4_-NH_2_ (**e**) and C 1s band of the adsorbed organic molecules and Prussian blue in the PB-MnFe_2_O_4_-NH_2_ (**f**).

To confirm the existence of a manganese ferrite structure and the presence of a Prussian blue coating, XPS measurements were performed. The XPS spectra of the MnFe_2_O_4_-NH_2_ sample identified peaks of iron, manganese, oxygen, nitrogen, and carbon (Fig. 5a). The high-resolution Mn 2p spectrum contained two characteristic peaks at 641.2 eV and 653.2 eV binding energies, which are characteristic for Mn 2p_1/2_ and Mn 2p_3/2_, confirming the presence of divalent^60^ Mn ions (Mn^2+^) in the MnFe_2_O_4_ spinel (Fig. 5b)^51,61^. The + 2 oxidation state of manganese is also supported by the difference in the positions of the two peaks resulting from the cleavage of the Mn 3s band (SI Fig. 1). The Mn 3s bands show an intensity maximum at binding energies of 88.7 eV and 82.6 eV, with a difference (ΔE) of 6.1 eV, supporting the presence of Mn(II)^62^. The Fe 2p spectra of the non-modified amine-functionalized manganese ferrite and its Prussian blue-coated counterpart show the Fe 2p_1/2_ and Fe 2p_3/2_ multiplets at 724.6 and 711 eV binding energy values, respectively (Fig. 5c). These peaks, along with the satellites (at 733.2 and 719.1 eV), indicate that Fe is present in the form of trivalent (Fe^3+^)^51,63^.

The presence of divalent manganese (Mn^2+^) and trivalent iron (Fe^3+^) confirms the chemical composition of MnFe_2_O_4_. This is further confirmed by the iron to manganese ratio of 2.0 (16.6 (n/n)% Fe and 8.2 (n/n)% Mn), corresponding to the stoichiometric ratios in the manganese ferrite (Table 1.). It was confirmed by XRD results, which indicated the presence of manganese ferrite spinel only; no other metal oxides were found (Fig. 4).

In contrast, the Fe: Mn ratio for manganese ferrite modified with Prussian blue is 2.6 (15.3 (n/n)% Fe and 5.8 (n/n)% Mn) this iron excess being explained by the presence of iron(II) iron(III) octadecacyanide (Fe^III^_4_[Fe^II^(CN)_6_]_3_) and the magnetite phase, which was also identified by XRD measurement (Fig. 4).

Potassium was also measurable in the PB-MnFe_2_O_4_-NH_2_ sample at 0.7 (n/n)% amounts, due to the use of potassium hexacyanoferrate. A significant increase in nitrogen content (from 0.8 n/n% to 4.2 n/n%) is observed for the Prussian blue-modified manganese ferrite due to its nitrogen content (Table 1).

**Table 1:** Surface mole percent (n/n%) of the elements in the MnFe_2_O_4_-NH_2_ and MnFe_2_O_4_-NH_2_ PB.

(*n*/*n*)%	C 1s	Fe 3p	Mn 3p	*N* 1s	O 1s	K 2p	Fe 3p/Mn 3p
MnFe_2_**O**_**4**_**-NH**_**2**_	22.0	16.6	8.2	0.8	52.5	-	2.0
MnFe_2_**O**_**4**_**-NH**_**2**_ PB	26.5	15.3	5.8	4.2	47.5	0.7	2.6

The aforementioned mentioned nitrogen in 0.8 (n/n)% is found in the C-N bonds of the amine functional groups in the MnFe_2_O_4_-NH_2_ sample (Fig. 5D). In contrast, in the MnFe_2_O_4_-NH_2_ PB sample, an additional band with high intensity was observed, contributing to the higher nitrogen content (4.2 n/n%) of the sample due to the C ≡ N bonds of the Fe^III^_4_[Fe^II^(CN)_6_]_3_ (Fig. 5d).

**Fig. 6:**
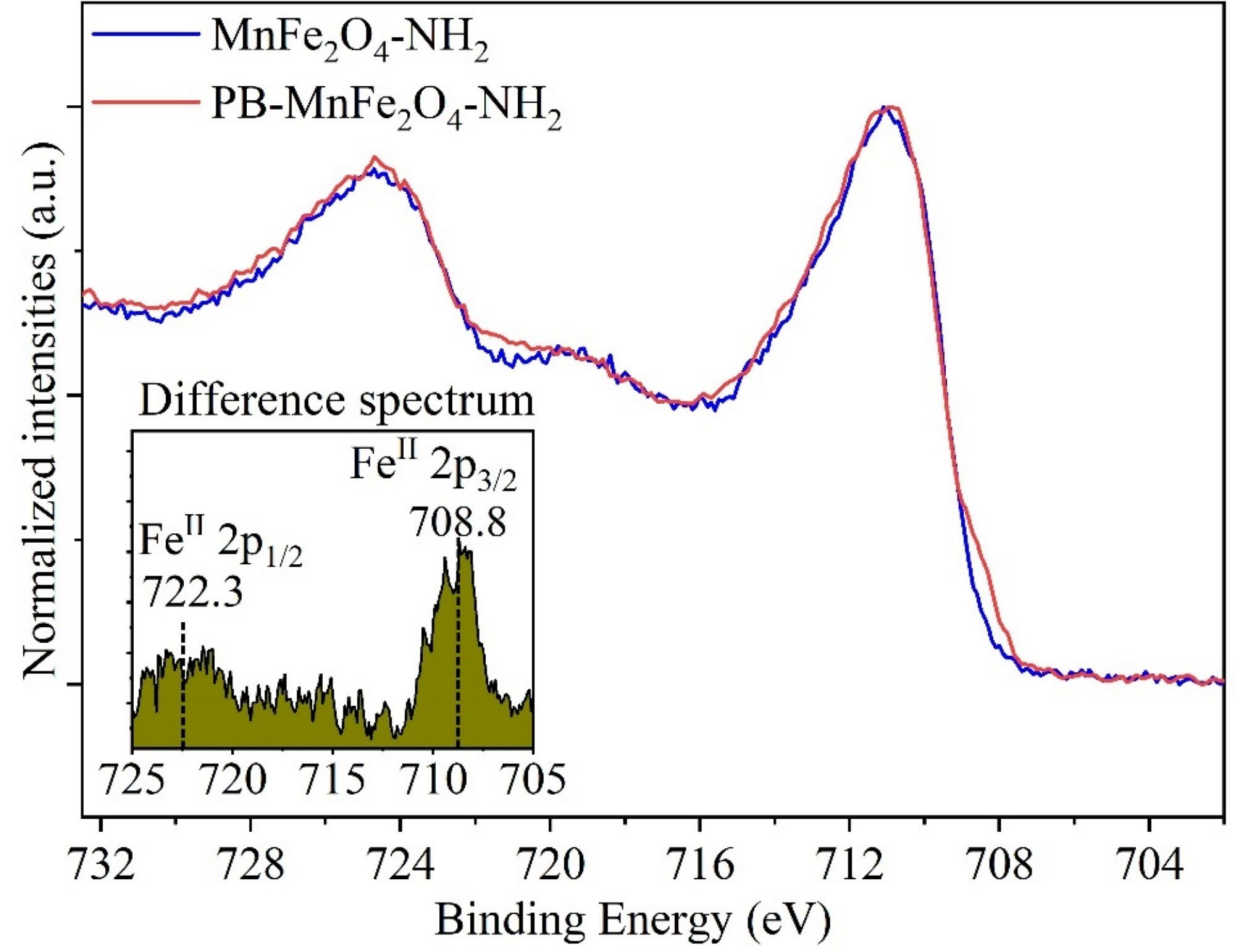
Difference X-Ray photoelectron spectrum of the Fe 2p spectra of the two manganese ferrite samples, indicates the presence of the divalent iron content of the Prussian blue in the PB-MnFe_2_O_4_-NH_2_ sample.

The oxygen contents of the two manganese ferrite samples were similar (52.5 n/n% and 47.5 n/n%), found as lattice oxide (at 530 eV), adsorbed water within the lattice (at 532.5 eV) and the hydroxyl groups of the adsorbed organic molecules (ethylene glycol) at 531.5 eV binding energy (Fig. 5e)^51^. The proportion of oxygen from the functional groups of organic compounds is about 6.3 (n/n)%, with the remaining 46.2 (n/n)% in metal oxides (and adsorbed water) in the case of the MnFe_2_O_4_-NH_2_ sample. The carbon content (22.0 n/n%) of the MnFe_2_O_4_-NH_2_ origin from the adsorbed ethylene glycol, ethanol amine (Fig. 5f) and, loosely bound adventitious carbon. On the C 1s spectra of the Prussian blue coated sample are identified peaks at 284.8 eV, 286.1 eV, 287.6 eV and 288.6 eV binding energy values, characteristic of the C-C, C ≡ N, C-OH, C-O-C, C = O and O-C = O bonds. These originate from the adsorbed organic species, including the adventitious carbon and the Prussian blue (Fig. 5f)^51^. The presence of K 2p_3/2_ and K 2p_1/2_ peaks at 293.2 eV and 295.9 eV is due to the presence of potassium hexacyanoferrate, K_4_[Fe(CN)_6_]^64^. The Fe(II) content of the Prussian blue in the PB-MnFe_2_O_4_-NH_2_ sample can be determined from the XPS spectrum by obtaining the difference spectrum of the Fe 2p spectra of the two manganese ferrite samples (Fig. 6)^65^.

**Fig. 7:**
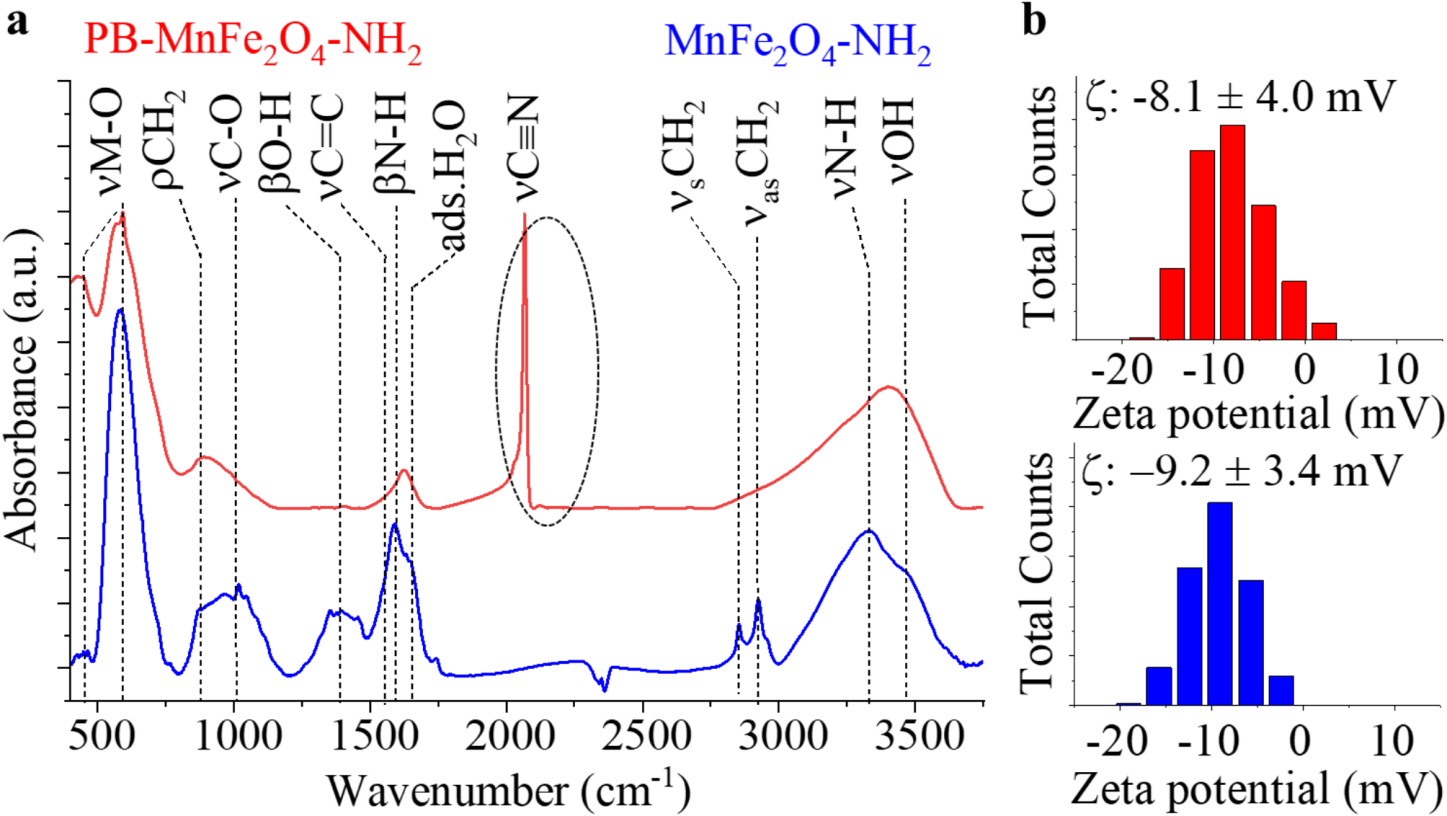
FTIR spectrum (**a**) and zeta potential distribution (**b**) of the MnFe_2_O_4_-NH_2_ (marked blue) and PB-MnFe_2_O_4_-NH_2_ nanoparticles (marked red). The C≡N bond characteristic of Prussian blue is marked with a circle on the FTIR spectrum.

The functional groups on the surface of the amine-functionalized manganese ferrite nanoparticles were identified by Fourier transform infrared spectroscopy (FTIR) (Fig. 7a). In the FTIR spectra, two bands are found, which contribute to the tetrahedral at 587 cm^− 1^ and octahedral complexes at 458 cm^− 1^ of the metal-oxygen bond vibrations in the spinel structures. The band at 587 cm^− 1^ was assigned to the vibrations of the Fe^3+^–O^2‒^. The peak at 458 cm^− 1^ represented the trivalent metal-oxygen vibration at the octahedral B-sites in the MnFe_2_O_4_-NH_2_ sample. The bands of these M-O vibrations are found in the FTIR spectra of another ferrites (CoFe_2_O_4_, Fe_3_O_4_, MgFe_2_O_4,_ NiFe_2_O_4_)^66–68^. The bands between 890 cm^− 1^ belong to -CH_2_ deformation vibration due to the presence of the adsorbed organic compounds, ethanol amine or ethylene glycol from the solvothermal synthesis of the MnFe_2_O_4_. The νC-O stretching vibration of the alcoholic groups originates from the adsorbed ethylene glycol, similar to the βOH band at 1390 cm^− 1^ and νOH band at 3481 cm^− 1^. The presence of the bands at 1627 cm^− 1^ and 3320 cm^− 1^ wavenumbers originates from the bending and stretching vibration of the amine functional groups. The C = C stretching vibration band was identified at 1583 cm^− 1^, originating from the adsorbed organic molecules. The presence of the adsorbed water molecules leads to a vibration band at 1662 cm^− 1^. The two low-intensity peaks at 2850 cm^− 1^ and 2930 cm^− 1^ were the symmetric and asymmetric stretching vibration modes of the aliphatic and aromatic C–H bonds. The hydroxyl and amine functional groups on the surface of the magnetic nanoparticles contribute to their good dispersibility in polar solvents, such as water. The protonation and deprotonation processes of the -NH_2_ and -OH groups contribute to the change in the surface charge of the nanoparticles, thereby affecting their zeta potential. The measured zeta potential in distilled water was − 9.2 ± 3.4 mV (Fig. 7b). In the case of the PB-MnFe_2_O_4_-NH_2_ sample, the presence of Prussian blue was confirmed by FTIR measurement. The spectrum showed the characteristic stretching vibration of CN bonds in the Fe^2+^–CN–Fe^3+^ fragment at 2065 cm^− 1^ wavenumber, alongside the previously detailed vibration bands^69–72^. CN bonding leads to a sharp and intense band in the FTIR spectrum, similar results have been obtained for magnetite particles treated with Prussian blue^28^. In our experiment, the Prussian blue coating on the surface of the magnetic nanoparticles was subsequently complexed, whereas Jomma et al. produced magnetite doped with Prussian blue by one-step solvothermal synthesis^73^. In their experiment, potassium hexacianoferrate was added to an ethylene glycol solution of Fe(III) chloride and then heated in an autoclave at 190 °C for 12 h. and K_3_Fe(CN)_6_, the above-mentioned νC ≡ N band of high intensity appeared in the infrared spectrum of the sample, confirming the presence of Prussian blue.

The average zeta potential of the PB-MnFe_2_O_4_-NH_2_ was − 8,1 ± 4, mV, very similar to the Prussian blue-free manganese ferrite (Fig. 7b). Due to the moderate zeta potential, electrostatic stabilization is not sufficient for the colloidal system to remain stable for a long time to ensure colloidal stability, steric stabilization was applied using polyvinyl pyrrolidone (PVP).

**Fig. 8:**
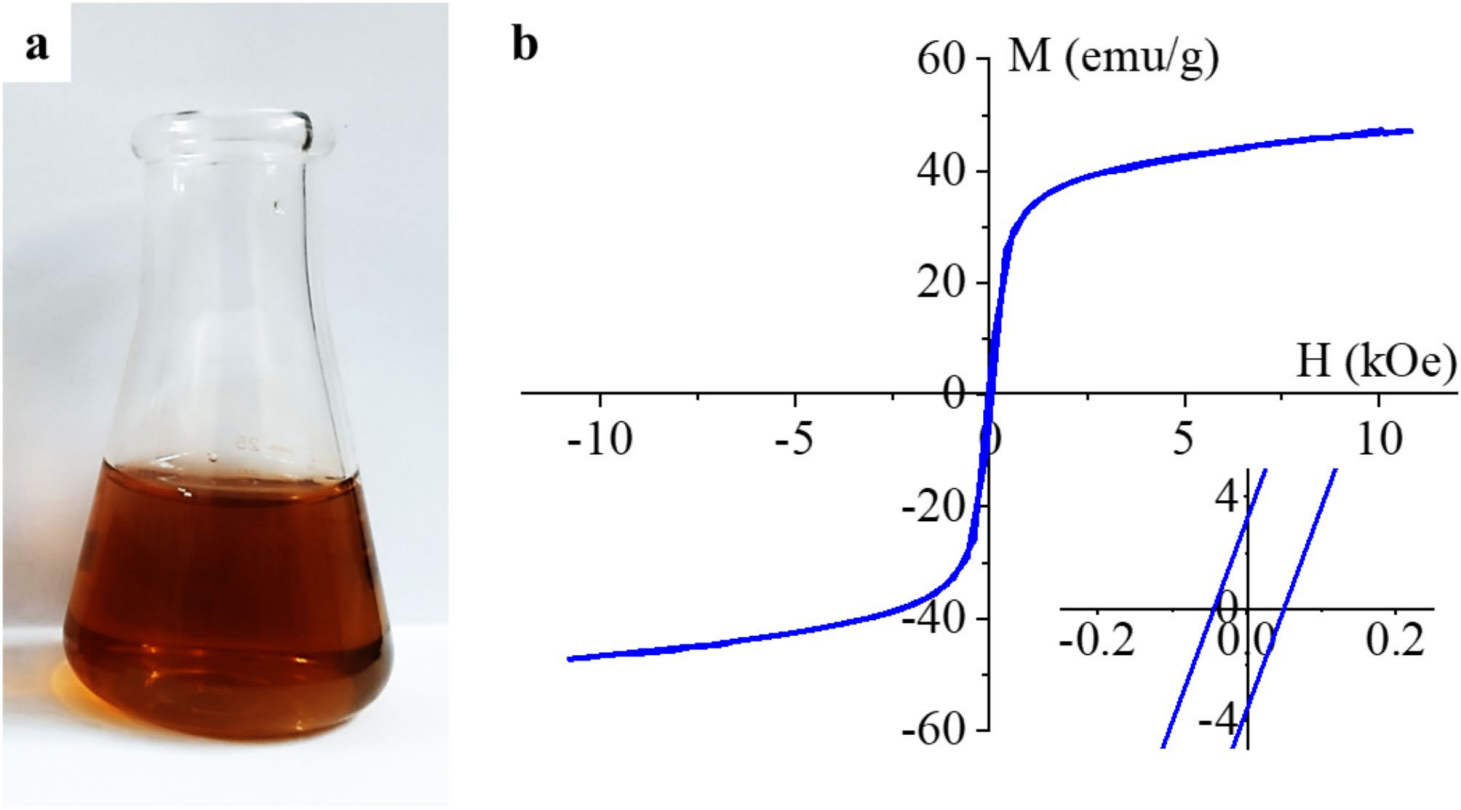
Dispersibility in water (**a**) and VSM curves (**b**) of the amine-functionalized MnFe_2_O_4_ nanoparticles. The magnified section of the VSM curve shows the hysteresis, i.e. the ferromagnetic property of the particles.

Owing to the presence of PVP, the manganese ferrite nanoparticles are well dispersible in water (Fig. 8a).

The magnetization curve of the ferrite sample was measured at 303 K for a magnetic field of 11 kOe using a vibrating-sample magnetometer (VSM). The magnetic saturation (Ms) reached 44 emu/g (Fig. 8b). This value is smaller than the reported magnetization for bulk MnFe_2_O_4_ (82 emu/g). The discrepancy originates from the enhanced surface-to-volume ratio of nanoparticles, where the canted surface spins do not contribute to overall magnetization^74^. The magnetization curve shows a very small hysteresis loop with low coercivity (Hc) and low remanent magnetization (Mr), as seen in the inset of Fig. 8b. The values of Hc (52 Oe) and Mr (3.7 emu/g) for the MnFe_2_O_4_-NH_2_ sample are small, indicating the soft ferromagnetic nature at room temperature (Fig. 8b). Manganese ferrite with similarly soft ferromagnetic properties (Ms: 65.8 emu/g, Mr: 8.9 emu/g and Hc:70 Oe) was prepared by Gao et al., with particle sizes in the 20–30 nm range^75^. Their synthesis method was different from ours, they used an ammonia coprecipitation procedure from a solution of iron(III) chloride and manganese(II) chloride, after hydrothermal treatment was carried in distilled water at 180 °C. The coprecipitation procedure supplemented with the above-mentioned hydrothermal treatment was applied by Karaagac et al., by varying the temperature of the hydrothermal reaction between 100 and 220 °C, with a synthesis time of 4 h^76^. As the synthesis temperature increased, Ms values increased from 35 to 64 emu/g, Hc from 22 to 58 Oe and Mr from 1.6 to 8.5 emu/g. This indicates that it is easier to magnetize the nanoparticles synthesized at higher temperatures, we carried out the synthesis at 198 °C, at the boiling point of ethylene glycol, at atmospheric pressure and obtained 44 emu/g Ms at 52 Oe coercivity, these values fit well into the ranges mentioned above.

The magnetization properties of manganese ferrite samples prepared by different synthesis methods are summarized in Table 2. It is observed that, the specific magnetization ranges of the MnFe_2_O_4_ from ~ 16 emu/g to ~ 78 emu/g, are associated with the particle size and crystal structure (Table 2).

**Table 2:** Comparison of the coercivity, saturation magnetization, and particle size of the different ferrite nanoparticles.

Sample ID.	Synth. method	d (nm)	Ms (emu/g)	Mr (emu/g)	Hc (Oe)	Ref.
MnFe_2_O_4_	Solvothermal	43	44	3.7	52	This work
S100	Hydrothermal	16.1	34	1.6	22	^76^
S120		15.4	49	3.0	28	
S1		1.8	16	0.1	1.0	
MnFe_2_O_4_	Hydrothermal	23	65.8	8.9	70	^75^
MF	sol–gel auto-combustion process	35	78.2	31.6	56.1	^77^
MnFe_2_O_4_	thermal decomposition method	10.4	33	0	0	^78^
MnFe_2_O_4_	hydrothermal method	20–30 nm	43.89	0	0	^79^
P0	thermal decomposition	6.5	26.6	0	0	^80^
MnFe_2_O_4_	co-precipitation	4.7	55	0	0	^81^
MnFe_2_O_4_	Solvothermal method	200–400	75	1.2	12	^82^
MnFe_2_O_4_	co-precipitation	5	69	0	0	^83^
MnFe_2_O_4_ (573)	thermal decomposition	11.2	72	0.1	0.7	^84^
MFNP_3_	co-precipitation technique	16.1	15.9	1.7	94.1	^85^
MFNP_5_		14.4	14.3	1.6	95.2	
MFNP_7_		11.5	14.8	1.6	93.7	
MnFe_2_O_4_	ball milling technique	8.4	41	0	0	^86^

**Fig. 9:**
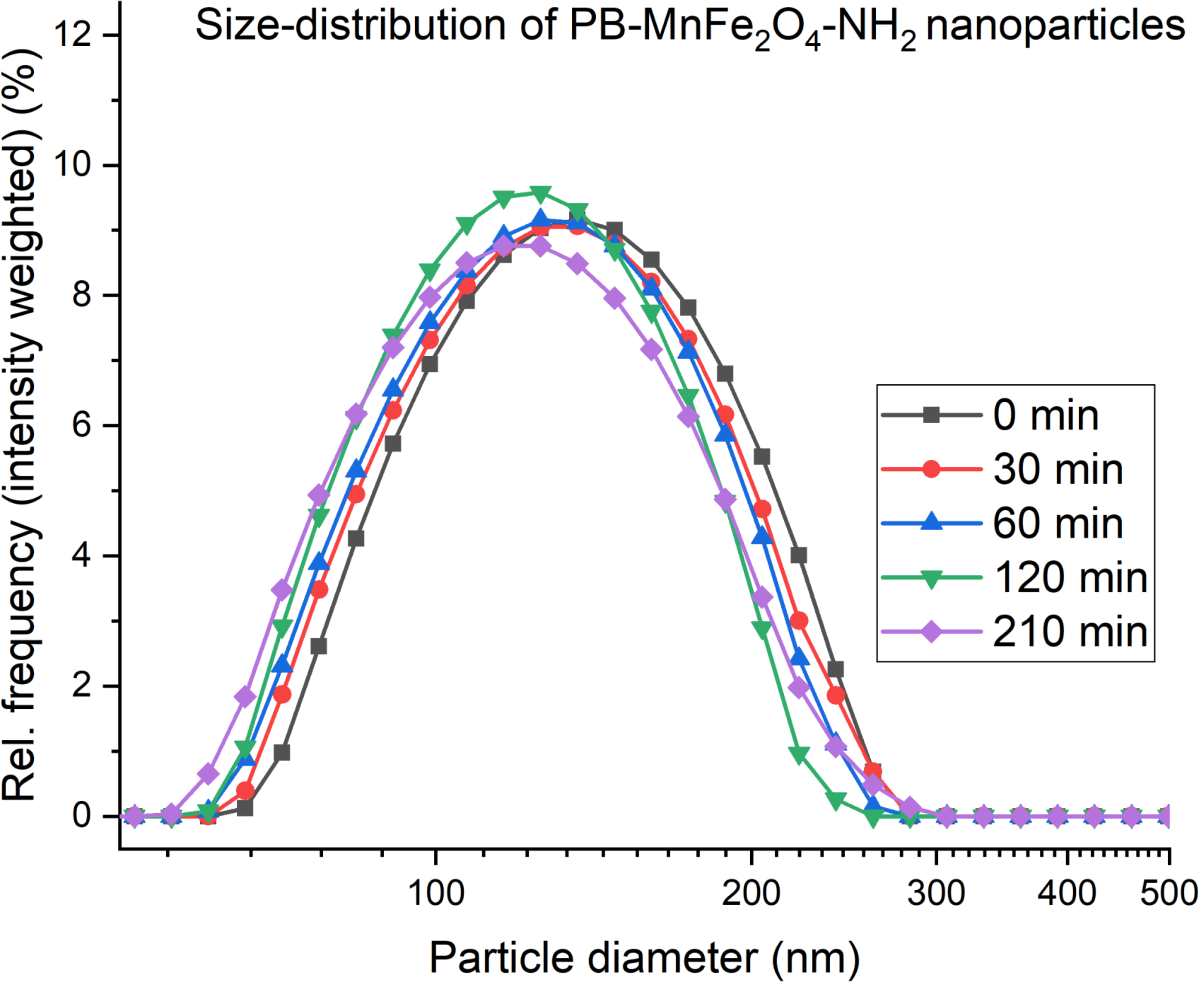
The size-distribution shift over time in the PB-MnFe_2_O_4_-NH_2_ samples. No significant change is visible over the measured time.

DLS tests were carried out in distilled water at pH 6.1 to study the colloidal stability of the PVP-stabilized nanoparticles. For the measurement, a 10 mg sample (PB-MnFe_2_O_4_-NH_2_ particles dried in PVP) was dispersed in 2 mL distilled water. The PB-MnFe_2_O_4_-NH_2_ samples were colloidally stable. DLS estimated the mean hydrodynamic diameter (intensity-based harmonic average) of PB-MnFe_2_O_4_-NH_2_ to be 140 ± 2 nm (average ± SD), which had only marginally altered over time. The results indicate that there was no substantial change in the colloidal phase over the 3.5 h of testing. The PB- MnFe_2_O_4_-NH_2_ nanoparticles did not flocculate or aggregate, as indicated by the computed 0.163 ± 0.018 summarized polydispersity index (PDI) (Fig. 9.). The above tests were necessary to ensure that the colloid remained stable for the 3 h prior to use, and our tests confirmed this. The contrast agent, when dried in PVP carrier, retains its stability for a very long time, which can be dissolved immediately and rapidly before polymer use, this provides steric stability for the nanoparticles dispersed in the aqueous medium. The chains of polymer molecules adsorbed on the surface of the particles prevent the nanoparticles from approaching each other and aggregating within range of distance for Van der Waals force.

### Test results of PB-MnFe_2_O_4_-NH_2_ contrast agent in magnetic resonance imaging

The purpose of in vitro measurements is to obtain approximate information about how a sample behaves in solution in a high magnetic field before in vivo measurements. From this measurement, theoretical calculations can be made to obtain the relaxivity of the samples, which is a good predictor of the sequence in which the material will be used (positive-illuminating or negative-darkening contrast agent). In vitro MRI measurements were performed on five different ferrite concentrations (0.01, 0.02, 0.05, 0.1, 0.2, 1 mg/mL). The Prussian blue-coated manganese ferrite samples were examined.

#### In vitro MRI measurements

The aim of the in vitro MRI measurements was to determine the capabilities of the PB-MnFe_2_O_4_-NH_2_ PVP to be used as an MRI contrast agent. The essential property of MRI contrast agents is relaxivity, the ability to modify the relaxation time (T1, T2 and T2*) of the medium per unit concentration. For this, a standardized method was used, previously described in our published work^87,88^.

Five PB-MnFe_2_O_4_-NH_2_-PVP samples with different ferrite concentrations (0.01, 0.02, 0.05, 0.1 and 0.2 mg/mL) were diluted in distilled water, poured in 2–2 mL Eppendorf tubes and placed in a sample holder. Compared to the previously described CuFe_2_O_4_-NH_2_ and ZnFe_2_O_4_-NH_2_, the PB-MnFe_2_O_4_-NH_2_ samples produced a similarly homogeneous MRI signal, as particles exceeded excellent colloidal stability during the measurements.

**Fig. 10:**
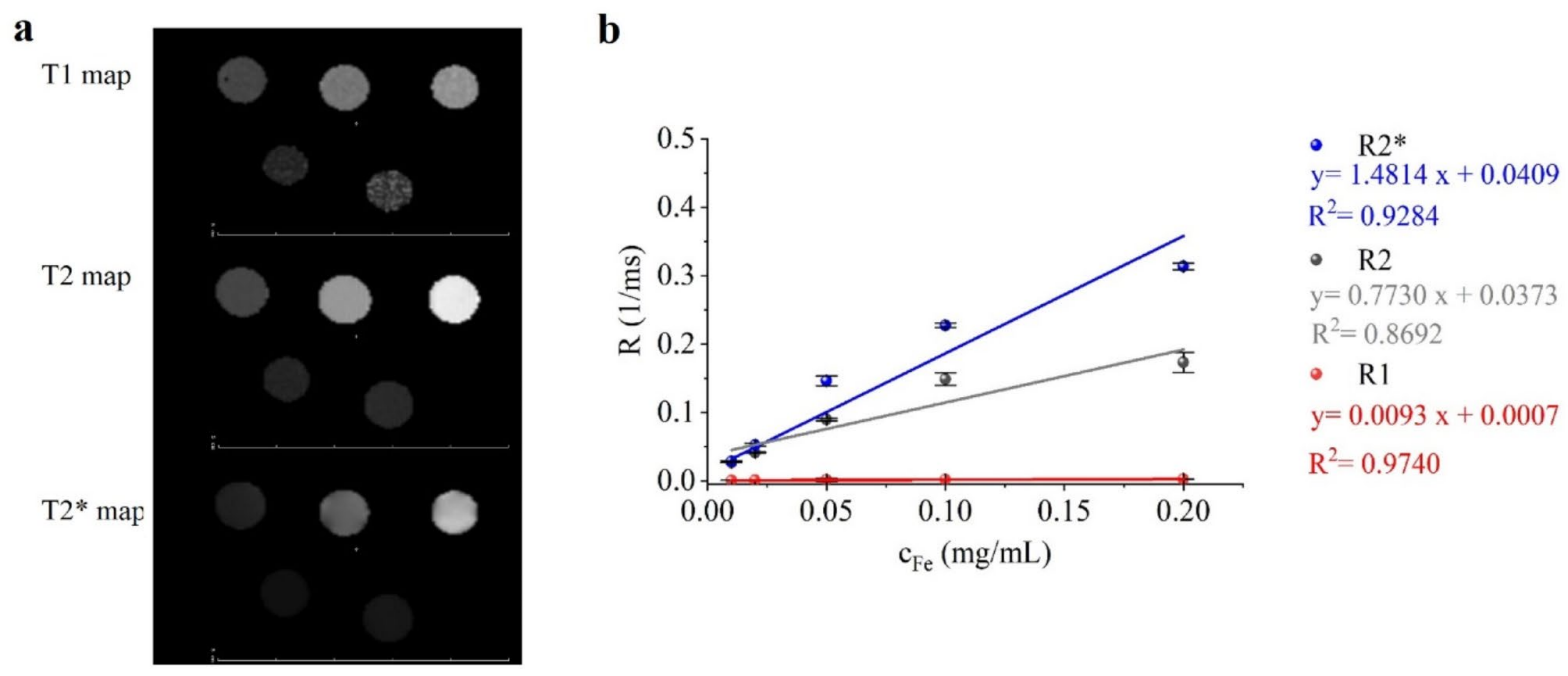
Voxel wise calculated relaxation time maps of PB-MnFe_2_O_4_-NH_2_-PVP concentration row (Panel** a**), from which transversal and longitudinal relaxation rates were determined for every ferrit concentration (Panel **b**). Solid lines show the result of the linear fit of relaxivities.

T1, T2 and T2* maps were calculated based on the Multi-IR FSE scan, the Multi-echo SE scan and the Multi-echo GRE scan respectively (Fig. 10a). The relaxivities of our sample were determined by the degree to which the relaxation rate (R1, R2, R2*), the inverse of the relaxation time, varies with ferrite concentration. Figure 10b depicts this linear relationship, and displays the fitted first-order polynomials (solid lines) to the measured relaxation rate values (dots). The goodness of the fit is characterized by the R2, the closer the value to 1 the more confident the fitted parameter.

The longitudinal relaxivity (r_1_) of the PB-MnFe_2_O_4_-NH_2_ sample was determined to be 0.0093 (mg/mL)^−1^ms^− 1^. The transversal relaxivity was measured also (r_2_: 0.7730 (mg/mL)^−1^ms^− 1^ and r_2_*: 1.4814 (mg/mL)^−1^ms^− 1^. In this sense, the Prussian blue-coated manganese-ferrite nanoparticles had similar characteristics to the previously examined ZnFe_2_O_4_-NH_2_, CuFe_2_O_4_-NH_2_ and other superparamagnetic iron oxide (SPIO) nanoparticles; their transverse relaxivities were in the same range as Feraheme and Endorem. Despite its PB content, the longitudinal relaxivity remained as low as it was in the case of ZnFe_2_O_4_-NH_2_, CuFe_2_O_4_-NH_2_.

The measured relaxivity values help to observe the r1/r2 ratio and determine the “true nature” of the contrast agent: the smaller the r1/r2, the more T2 effect is present in the sample. The r1/r2 ratios of our samples (0.012) were significantly lower than Endorem (80–150 nm) exhibiting an r1/r2 ratio of 0.044 and Resovist (62 nm) showing a ratio of 0.032 ^90^. This indicates that our ferrite solution functions predominantly as a T2 contrast agent with negligible T1 effects^90^.

#### Toxicology measurement

Alamar Blue is utilized to assess cellular redox potential and offers benefits over the MTT assay, primarily due to its simpler sample preparation process^91^. While there are some concerns about the assay’s biochemical mechanisms and interactions with non-porous silicon in the absence of cells, it is generally well-regarded for its sensitivity and reliability in various cell types^92^.

**Fig. 11:**
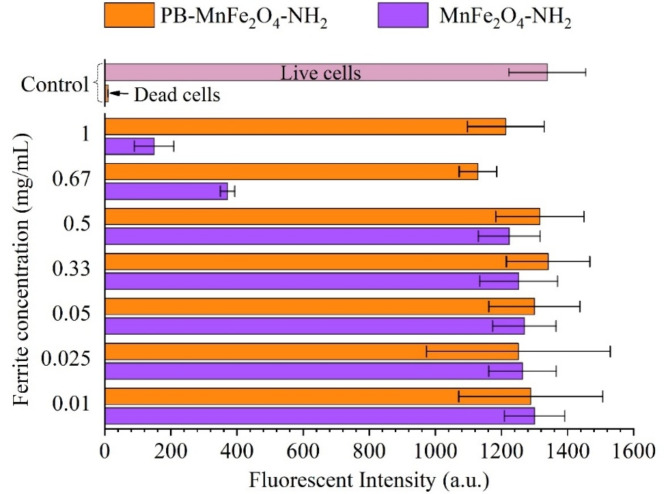
The results of the Alamar Blue fluorescent assay on HEK293 cells. Positive and negative control measurements were established to evaluate the validity of the results (red and pink columns). The conventional PVP coated MnFe_2_O_4_-NH_2_ particles (blue columns) were compared to the PB-MnFe_2_O_4_-NH_2_ samples (orange columns). Clearly, PB coating reduced the toxic effects of MnFe_2_O_4_-NH_2_ particles in case of higher than 0.5 mg/mL.

To assess the efficacy of the PB coating on MnFe_2_O_4_-NH_2_ particles, the Alamar Blue assay was employed. HEK293 cells were cultured in DMEM and water media for 24 h as control conditions (Fig. 11). The rationale for using water as a medium is to simulate a worst-case scenario for cellular viability, as water lacks essential nutrients, growth factors, and osmotic balance, leading to rapid cell death. This contrasts with DMEM, which provides a nutrient-rich environment that supports cell growth and maintains cellular homeostasis. The nanoparticles, whose toxicity is not well understood, were tested in these conditions to observe their effects on cell viability. PVP and PB-coated MnFe_2_O_4_-NH_2_ particles were tested at concentrations ranging from 1 mg/mL to 0.1 mg/Ml. Control measurements indicated a significant difference between cells cultured in DMEM, which supports cell growth, and those in water, which induces cell death. The PB coating on MnFe_2_O_4_-NH_2_ particles markedly influenced the viability of HEK293 cells compared to the traditional PVP coating which was evidenced by higher RFU values at all tested ferrite concentrations, directly correlating with increased cell viability.

#### In vivo MRI measurement

**Fig. 12:**
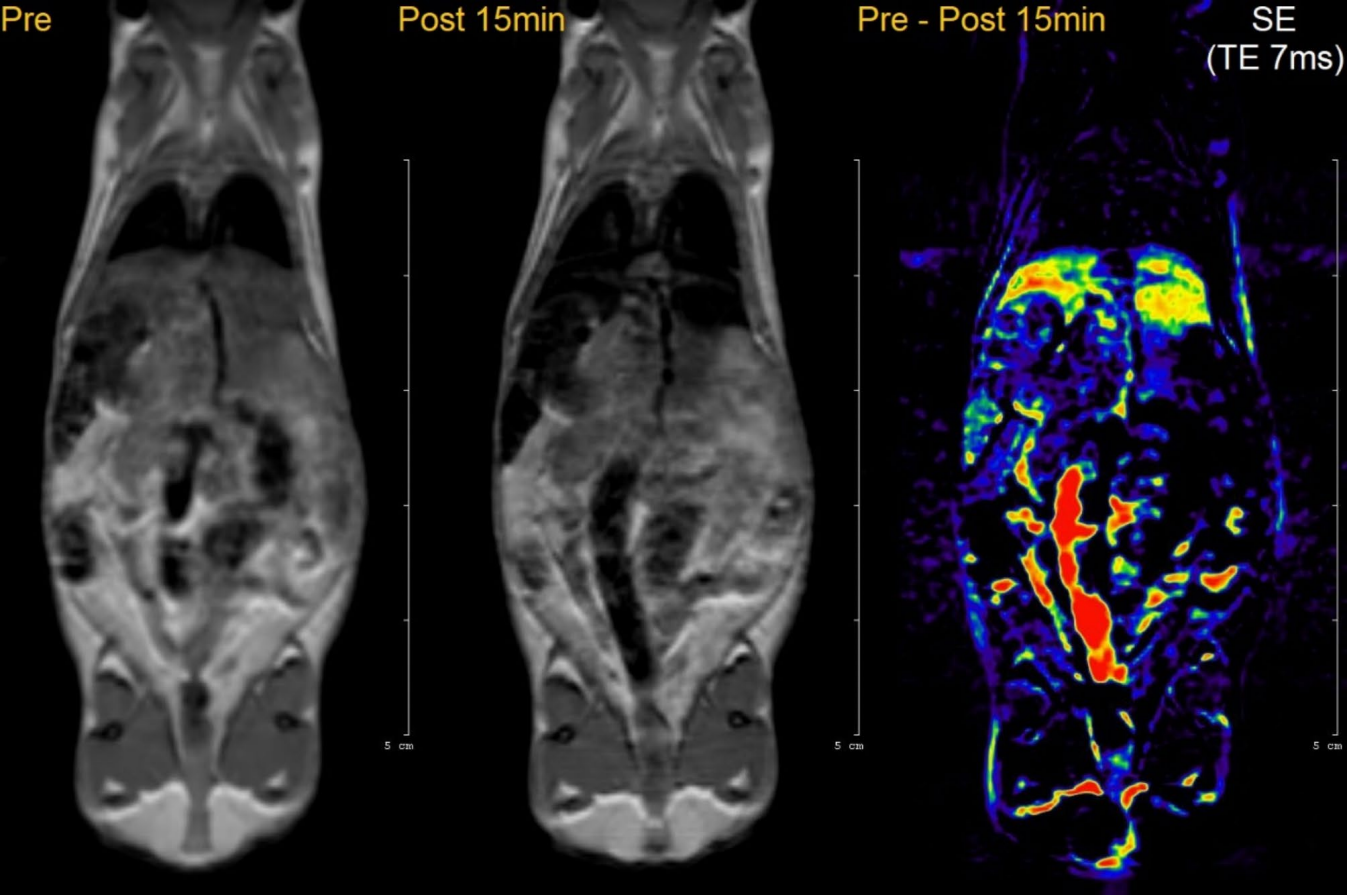
T2-weighted spin-echo (SE) scans of a mouse at 2 different time points—before injection and 15 min after intravenous injection of PB-MnFe_2_O_4_-NH_2_. The voxel-wise change of SE signal intensities (3rd image) shows the highest uptake in the liver. Hyperintense changes are also visible on the subtraction image, in the abdominal aorta, kidney and spleen.

PB-MnFe_2_O_4_-NH_2_ sample was chosen to be injected in vivo due to its stability in vitro measurements. A concentrated sample with a ferrite concentration of 1 mg/mL was available for injection; a dose of 6.55 mg/kg body weight was intravenously injected into the tail vein as a 0.20 mL bolus. An immediate uptake was observed in the liver and the spleen (Fig. 12) as on the post-15 min spin echo scan the signal intensities decreased compared to the pre-scan. This is confirmed by the voxel-wise calculation of the signal intensity change on the SE scan between the Pre and Post 15-min scans (right section of Fig. 12). The highest change comes from bowel motion (red area) and besides that the nanoparticle accumulation in the liver (yellow area). This suggests that the PB-MnFe₂O₄-NH₂ particles are rapidly taken up by the liver and spleen, highlighting their potential for targeted delivery and imaging applications. No other organs accumulated the nanoparticles according to our spin-echo (SE) scans. Compared to results including Prussian Blue nanoparticles, the predominant T2 and T2* contrast of the ferrite nanoparticles can be detected in the figures. The inverse signal increase is noticeable in the kidney area, whereas Prussian Blue nanoparticles would exhibit hyperintense changes throughout the lungs, kidneys and liver^27,93,94^.

## Methods

### Materials

The manganese ferrite nanoparticles were synthesized from the following ingredients: manganese (II) nitrate tetrahydrate, Mn(NO_3_)_2_·4H_2_O (Carl Roth GmbH, Karlsruhe, Germany); iron(III) nitrate-nonahydrate, Fe(NO_3_)_3_·9H_2_O (VWR International, Leuven, Belgium). Ethylene glycol, HOCH_2_CH_2_OH, (VWR Int. Ltd., F-94126 Fontenay-sous-Bois, France); monoethanolamine, NH_2_CH_2_CH_2_OH (Merck KGaA, D-64271 Darmstadt, Germany); and sodium acetate, CH_3_COONa (ThermoFisher GmbH, D-76870 Kandel, Germany) were used as reducing agents and dispersion media for the metal precursors. Potassium hexacyanoferrate(II) trihydrate, K_4_[Fe(CN)_6_]·3H_2_O and iron(III)-chloride (anhydrous), FeCl_3_ (Sigma Aldrich Ltd., MO 63103, Saint Louis, USA) were used for deposition of Prussian blue layer on the surface of the manganese ferrite nanoparticles.

### Characterization techniques

#### Analysis of particle size and morphology with applying of transmission electron microscopy

For characterization of the particle size, morphology and crystalline phases of the manganese ferrite nanoparticles were carried out by high-resolution transmission electron microscopy (HRTEM). For the HRTEM examination, a Talos F200X G2 electron microscope with field emission electron gun, X-FEG (accelerating voltage 20–200 kV) was used. For imaging and selected area electron diffraction (SAED) measurements, a SmartCam digital search camera (Ceta 16 Mpixel, 4k x 4k CMOS camera) was used with a high-angle annular dark-field (HAADF) detector. During the HRTEM examination, the aqueous dispersion of ferrite was dropped on 300 mesh copper grids (Ted Pella Inc., 4595 Redding, CA 96003, USA).

#### Identification of the crystal phases present in the sample mean of the X-ray diffraction method

Identification and quantification of the crystal phases were carried out by X-ray diffraction (XRD) measurements using a Bruker Discovery diffractometer (Cu-Kα source, 40 kV and 40 mA) in parallel beam geometry (Göbel mirror) with a Vantec detector using Powder Diffraction Files (PDFs). The average crystallite size of the manganese ferrite domains was calculated using the mean column length calibrated method, employing full width at half maximum (FWHM) and the width of the Lorentzian component of the fitted profiles (for the evaluation, TOPAS 4 software was applied).

#### Identification and quantification of the PB in the samples by X-ray photoelectron spectroscopy

To determine the composition and chemical state of the sample surfaces, XPS measurements were performed using a Thermo Scientific ESCALAB Xi^+^ instrument. A monochromatized Al K-alpha source (1486.6 eV) with a 650 μm spot size was used. Due to the ferromagnetic nature of the samples, the electron transfer lens of the instrument was used in electrostatic mode. For each sample, wide-range spectra were collected (at an analyzer pass energy of 80 eV) to survey the elemental composition. For quantitative and chemical state analysis, high-resolution spectra (at 20 eV pass energy) were recorded for the following photoelectron lines: C 1s, O 1s, Mn 2p, Fe 2p, N 1s, Mn 3s, Fe 3p regions. Charging of the sample surface was compensated using the instrument’s automatic built-in dual charge compensation system. The energy of sp3-bonded carbon (C-C/C-H) in C 1s, set at 284.8 eV was used as an internal reference for fine energy scale adjustment. All elemental composition calculations were performed assuming that the samples were homogeneous within the XPS-detected volume. The errors in the quantitative analysis (elemental composition) were estimated to be in the range of ± 10%.

#### Identification of the functional groups on the surface of manganese ferrite nanoparticles

The manganese ferrite samples were examined with Fourier transform infrared spectroscopy (FTIR) using a Bruker Vertex 70 spectroscope in transmission mode to identify their surface functional groups. For the FTIR measurements, 15 mg of the sample was pelletized with 250 mg of spectroscopic grade KBr.

#### Characterization of the magnetic behaviour of the manganese ferrite by vibrating-sample magnetometer

The magnetic characterization of ferrite nanoparticles was carried out using a self-developed (University of Debrecen) vibrating-sample magnetometer (VSM) system based on a water-cooled Weiss-type electromagnet. The powder samples were pelletized for measurements, with a typical mass of 20 mg. The magnetization (M) was measured as a function of the magnetic field (H) up to a field strength of 10,000 Oe at room temperature.

#### Measurements of the hydrodynamic diameters of the nanoparticles by dynamic light scattering technique

For measurements of the hydrodynamic size, size distribution, and zeta potential, a Litesizer 500 (Anton Paar, Hamburg, Germany) was used. DLS (dynamic light scattering) measurements were performed at 25 °C in automatic mode (for backscatter detector, fixed at 175°; for side scatter, 90° detector angle; for front scatter, 15° detector angle) using a 633 nm He-Ne laser. Samples were measured in polystyrene disposable cuvettes (Anton Paar, Hamburg, Germany). The tests were carried out in distilled water. A 10 mg sample (PB-MnFe_2_O_4_-NH_2_ nanoparticles dried in PVP) was dissolved in 2 mL distilled water. The pH was 6.1.

#### Toxicology tests on manganese ferrite

The human embryonic kidney cell line (HEK 293; ATCC, Manassas, VA) was maintained in Dulbecco’s modified Eagle’s medium (DMEM, Lonza) supplemented with 10% fetal bovine serum, 50 U/mL penicillin, and 50 µg/mL streptomycin in a 5% humidified CO_2_ incubator at 37 °C in cell culture flasks. For toxicology measurements, the cells were transferred onto black flat-bottom chimney 96-well plates (Greiner Bio-One, ref 655076) at a density of 10,000 cells/well, where they were allowed to adhere for 24 h. PVP and PB-coated MnFe_2_O_4_-NH_2_ were added to the cells at ferrite concentrations ranging from 1 mg/mL to 0.01 mg/mL. Water and DMEM were used as positive and negative controls, respectively. After 24 h of incubation at 37 °C in a 5% humidified CO_2_ incubator, the DMEM medium was exchanged for a transparent medium. To assess the toxicology of the MnFe_2_O_4_-NH_2_ nanoparticles, Alamar Blue (Invitrogen) was added at a ten-fold dilution, and the cells were incubated for 2 h at 37 °C^95^. Fluorescence intensity was detected using a Varioskan Lux multimode microplate reader (Thermo Fisher Scientific, Waltham, MA, USA) at room temperature, with an excitation wavelength of 570 nm and an emission wavelength of 590 nm. In the case of every well, three parallel measurements were conducted. Results are shown as relative fluorescence units (RFU).

#### Magnetic resonance imaging

MRI measurements were performed in vitro using a NanoScan PET/MR system (Mediso, Budapest, Hungary), equipped with a 3 T magnetic field, 600 mT/m gradient system, and a volume transmit/receive coil with a diameter of 72 mm for in vitro samples and 42 mm for mouse scans. In vitro scans were performed on five different manganese ferrite concentrations (0.01, 0.02, 0.05, 0.1 and 0.2 mg/mL) each held in a 2 mL Eppendorf tube in an in-house made sample holder. All FSE scans and relaxometry measurements were performed with the same geometrical parameters. One coronal slice was imaged with a slice thickness of 4 mm, a field of view of 50 mm, and an in-plane resolution of 0.36 mm. To determine T1 relaxation times, a Multi-IR FSE 2D sequence was used, with a repetition time of 5200 ms, an echo time of 5.8 ms, and inversion times of 100, 400, 600, 800, 1000, 1300, and 2000 ms. The acquisition time was 21 min.

The T2 relaxation times were determined using a Multi-echo SE 2D sequence with a repetition time of 3856 ms and a first echo time of 5.5 ms, followed by 32 echoes with echo spacing intervals of 5.55 ms. The measurement time was 9 min. A Multi-echo GRE 2D sequence was used for the calculation of T2* relaxation times, with a repetition time of 350 ms and a shortest echo time of 1.71 ms, followed by 32 echo spacing with echo intervals of 1.91 ms; the acquisition measurement time was 5 min. Based on these scans T1, T2 and T2* relaxation times were calculated voxel-wise by Fusion software (Mediso, Budapest, Hungary) and relaxation time maps were created.

An In vivo imaging study was performed on a healthy mouse (10-week-old female BalbC). The mice (INNOVO Ltd., Gödöllő, Hungary) were kept at 22 ± 3 °C; the relative humidity was 30–70%, and the light/dark cycle was 12/12 h. The animals were maintained on a standard rodent pellet diet (INNOVO Ltd., Gödöllő, Hungary) with tap water available ad libitum.

In vivo measurements were performed on mice under isoflurane anaesthesia (5% for induction and 1.5–2% to maintain the appropriate level of anaesthesia; (3% for induction and 1.5–2% to maintain the appropriate level of anaesthesia; Arrane, Baxter, Newbury, UK). 0.2 mL of the 1 mg/mL contrast material was injected into the tail vein of the animal, resulting in a 6.5 mg/bwkg dose of ferrite. To investigate the biodistribution of the ferrite nanoparticle a spin echo (SE) scan was repeated at three different time points (pre-injection, 15 min and 1 day post-injection). Imaging was acquired on 7 coronal slices with 80 × 50 mm FOV, in plane resolution of 0.36 mm, slice thickness of 0.8 mm, 2 averages and TR/TE 3 s/7 ms. Signal intensities on the images recorded at different timepoints were compared visually to determine which organ accumulates the ferrite nanoparticles. In the case of pre and 8 min, post scans voxel-wise subtraction was possible, because between these two scans mouse was not moved and maintained in anaesthesia.

### Ethics declarations and approval for animal experiments

For the in vivo measurements, *n* = 2 female, 10-week-old BalbC mice were used. 0.2 mL of the 1 mg/mL contrast material was injected into the tail vein of the animals. In vivo measurements were performed with the mice under isoflurane anaesthesia (5% for induction and 1.5–2% to maintain the appropriate level of anaesthesia (Arrane, Baxter, Newbury, UK)). All procedures were conducted in accordance with the ARRIVE guidelines (PLoS Bio 8(6), e1000412, 2010) and the guidelines set by the European Communities Council Directive (86/609 EEC). The study was approved by the Animal Care and Use Committee of Semmelweis University (PE/EA/01319-4/2023). We confirm that all experiments were performed in accordance with relevant guidelines and regulations.

### Synthesis methods of the manganese ferrite nanoparticle based contrast agent

#### Synthesis of the amine functionalized manganese ferrite nanoparticles

**Fig. 13:**
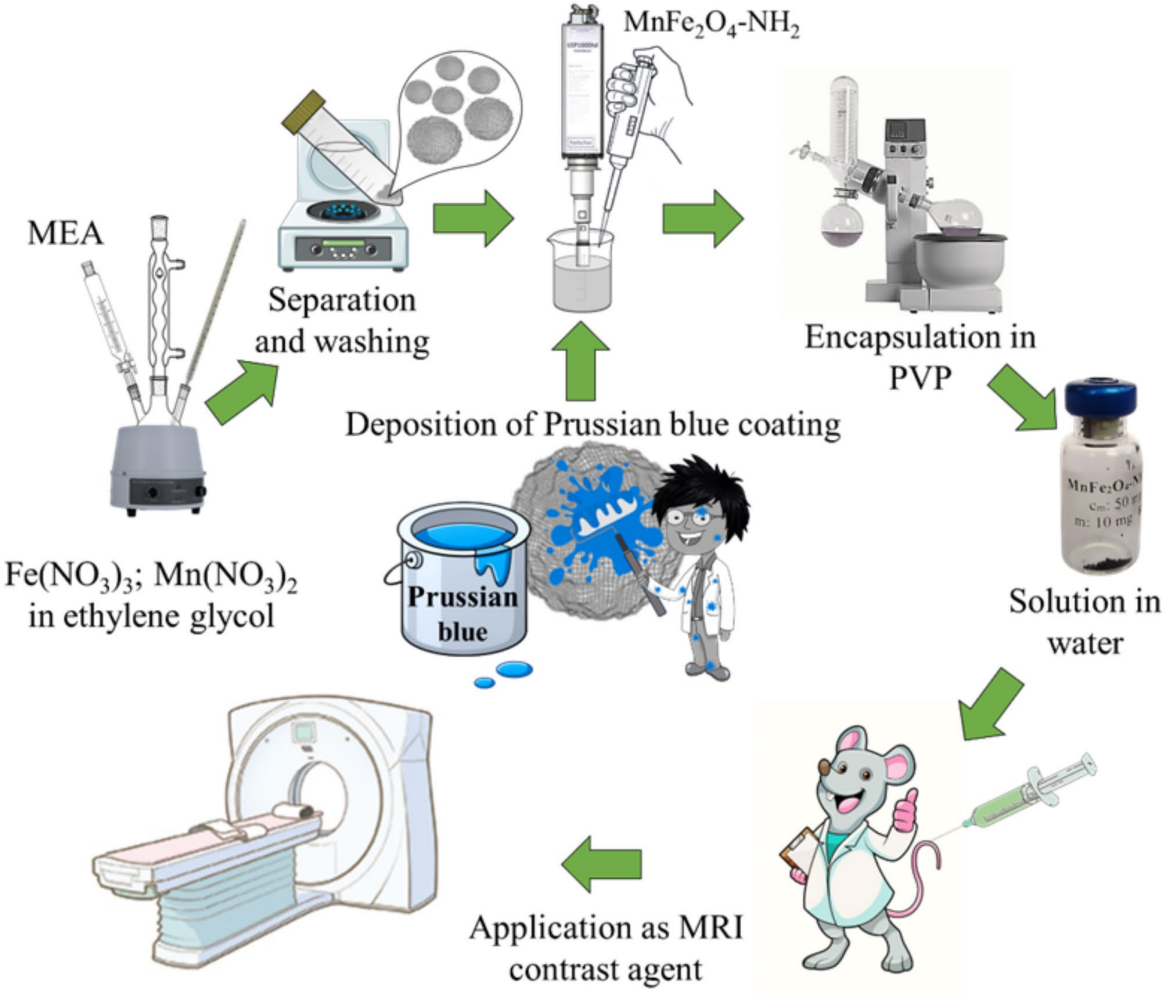
Schematic illustration of the synthesis and chemical modification of the manganese ferrite nanoparticles.

Amine-functionalized MnFe_2_O_4_ spinel nanoparticles were synthesized by a solvothermal method at atmospheric pressure at 198 °C under reflux for 12 h (Fig. 13). During this process, iron(III) nitrate nonahydrate (20 mmol) and manganese(II) nitrate tetrahydrate (10 mmol) were dissolved in 50 mL of ethylene glycol. Sodium acetate (12.30 g, 150 mmol) was dissolved in another 100 mL of ethylene glycol and heated to 198 °C in a three-necked flask under reflux and continuous stirring. The solution of the metal precursors was added to the glycol-based sodium acetate solution, followed by the addition of 35 mL of monoethanolamine (MEA). After 12 h of continuous agitation and reflux, the cooled solution was separated using a magnet. The solid phase was washed several times with distilled water and then dispersed in distilled water.

#### Deposition of Prussian blue on the surface of the amine-functionalized manganese ferrite nanoparticles

An aqueous colloid of 100 mg of amine-functionalized MnFe_2_O_4_ nanoparticles was added to 50 mL of an aqueous solution of 0.1 millimole potassium ferricyanide during ultrasonication (Fig. 13). After three minutes of sonication, a 10 mL solution of 0.1 millimole iron(III) chloride was added to the ferrite colloid during continuous sonication. After the above steps, a Prussian blue (Fe_4_[Fe(CN)_6_]_3_) layer formed on the surface of the manganese ferrite nanoparticles according to the overall reaction below:

$$\:4\:{FeCl}_{3}+\:{3\:K}_{4}[Fe\left({CN)}_{6}\right]={{Fe}_{4}[Fe\left({CN)}_{6}\right]}_{3}+\:12\:KCl$$

Afterwards, the magnetic nanoparticles were collected using a magnet and washed several times with distilled water. The magnetic phase was redispersed in a 100 mL solution of 1.80 g PVP using an ultrasound homogenizer. The water from the PVP-containing dispersion of manganese ferrite was evaporated using a rotary vacuum evaporator, and the solid phase was dried at 80 °C overnight.

## Conclusions

Amine-functionalized manganese ferrite magnetic nanoparticles were synthesized by the solvothermal method the magnetic particles were treated with potassium hexacyanoferrate as a complexing agent and Fe(III) chloride using high-power ultrasound. Due to the special reaction conditions of the ultrasonic synthesis method (owing to its intense cavitation force), it is a promising technique for depositing Prussian blue (Fe^III^_4_[Fe^II^(CN)_6_]_3_), as a biocompatible complex layer on the surface of manganese ferrite nanoparticles. The formation of the Prussian blue was confirmed by FTIR and XRD measurements. The morphological characteristics of the Prussian blue-coated and the untreated manganese ferrite samples were different the integrity of the spherical nanoparticles was reduced in the case of the Prussian blue-coated sample (MnFe_2_O4-NH_2_ PB), as the nanoclusters seen in the untreated manganese ferrite (MnFe_2_O_4_-NH_2_) samples were dispersed into small crystallites by the complexation effect, as confirmed by TEM studies. Differences were observed in the phase composition based on the XRD measurements. The untreated sample contained only the jacobsite (MnFe_2_O_4_) phase, but after treatment with potassium hexacyano ferrate, formed other compounds: magnetite (Fe_3_O_4_), Prussian blue (Fe^III^_4_[Fe^II^(CN)_6_]_3_). The zeta potential of the PB-MnFe_2_O_4_-NH_2_ was − 8.1 ± 4.0 mV, which was similar to the untreated MnFe_2_O_4_-NH_2_ sample + which had a zeta potential of + 9.2 ± 3.4 mV. Due to the low zeta potential of the nanoparticles, the stability of their aqueous colloidal systems was insufficient. To overcome the problem of colloidal stability, these ferrite nanoparticles were stabilized by polyvinylpyrrolidone in a dried form. PB-MnFe_2_O_4_-NH_2_ nanoparticles were embedded in PVP (ferrite content of 50 mg/g). The stability of the system was validated by DLS measurements, during which no significant change in the size distribution was observed. The synthesized particles have excellent stability in aqueous solutions, and their physical shape and size can be determined as part of the in vitro characterization. These polymer-stabilized red-brown crystals could be easily redispersed in distilled water and are applicable as contrast agents in magnetic resonance imaging (MRI).

In vitro MRI measurements allowed the determination of longitudinal and transversal relaxivities of PB-MnFe_2_O_4_-NH_2_-PVP nanoparticles based on the T1, T2, and T2* maps of a concentration row sample. It showed particularly high transversal relaxivity and low longitudinal relaxivity, which predicts a predominant T2 contrast, thus signal decrease on MRI images.

In vivo MRI in mouse showed an accumulation of intravenously administered PB-MnFe_2_O_4_ nanoparticles in the liver and spleen. This biodistribution – high uptake in the liver and slow wash out from there – is in agreement with the biodistribution of other superparamagnetic ferrite species. Nanoparticles with hydrodynamic size in the 100 nm range are taken up by the reticuloendothelial system (RES) in the liver.

Nevertheless, non-functionalized Prussian blue showed less significant longitudinal and transversal relaxivities, which did not support its in vivo use. On the other hand, the contrast capabilities of ferrites are mainly limited to signal decreasing on T2 and T2*-weighted images, in which the hypointense signal is not the desired one in every type of application. However, combining the two types of nanoparticles and exploiting their advantageous features; attaching them to other iron-based contrast materials, such as ferrites, could be a fundamental step in creating a new generation of iron-based contrast materials. Further optimization of the PB-MnFe_2_O_4_-NH_2_ nanoparticles, such as increasing longitudinal relaxivity (T1 contrast capability), drug molecule incorporation, and using them as a drug carrier system, could further increase the significance of such a system entering the market.

## Data Availability

The datasets used and/or analysed during the current study available from the corresponding author on reasonable request.

## References

[1] Manohar, A., Vijayakanth, V., Vattikuti, S. V. P. & Kim, K. H. A mini-review on AFe_2_O_4_ (A = Zn, Mg, Mn, Co, Cu, and Ni) nanoparticles: photocatalytic, magnetic hyperthermia and cytotoxicity study. *Mater. Chem. Phys.* **286**, 126117 (2022).

[2] Tietze, R. et al. Magnetic nanoparticle-based drug delivery for cancer therapy. *Biochem. Biophys. Res. Commun.* **468**, 463–470 (2015). 26271592 10.1016/j.bbrc.2015.08.022

[3] Prodělalová, J., Rittich, B., Španová, A., Petrová, K. & Beneš, M. J. Isolation of genomic DNA using magnetic Cobalt ferrite and silica particles. *J. Chromatogr. A*. **1056**, 43–48 (2004). 15595531

[4] Chen, C., Zheng, Z., Liu, C. & Yang, W. Synthesis of magnetic Fe_3_O_4_@Al^3+^ particles and its application in DNA extraction. *https://doiorg* **41**, 311–318 (2022). 10.1080/02726351.2022.2085217

[5] Comanescu, C. Magnetic nanoparticles: current advances in nanomedicine, drug delivery and MRI. *Chem* **4**, 872–930 (2022).

[6] Xu, J. et al. A one step method for isolation of genomic DNA using multi-amino modified magnetic nanoparticles. *RSC Adv.* **11**, 3324–3332 (2021). 35424297 10.1039/d0ra09409a PMC8693999

[7] Hikosaka, R., Nagata, F., Tomita, M. & Kato, K. Adsorption and desorption characteristics of DNA onto the surface of amino functional mesoporous silica with various particle morphologies. *Colloids Surf. B Biointerfaces*. **140**, 262–268 (2016). 26764114 10.1016/j.colsurfb.2015.12.054

[8] Sheng, W. et al. Amine-functionalized magnetic mesoporous silica nanoparticles for DNA separation. *Appl. Surf. Sci.* **387**, 1116–1124 (2016).

[9] Wan, S. et al. Dysprosium-Modified gold nanoparticles as T2exContrast agents for magnetic resonance imaging. *ACS Appl. Nano Mater.* **3**, 9433–9439 (2020).

[10] Dojcsák, D. et al. NH_2_-Functionalized magnetic nanoparticles for the N-Glycomic analysis of patients with multiple sclerosis. *Int. J. Mol. Sci.* **23**, (2022). 10.3390/ijms23169095 PMC9409089 36012360

[11] Ebrahimi, P. & Barbieri, M. Gadolinium as an emerging microcontaminant in water resources: Threats and opportunities. *Geosci.* Vol. 9, Page 93 9, 93 (2019). (2019).

[12] McDonald, J. S. & McDonald, R. J. MR imaging safety considerations of gadolinium-Based contrast agents: gadolinium retention and nephrogenic systemic fibrosis. *Magn. Reson. Imaging Clin. N Am.* **28**, 497–507 (2020). 33040991 10.1016/j.mric.2020.06.001

[13] Nasrin, S. et al. Study of the suitability of manganese-substituted Cobalt ferrites nanoparticles as MRI contrast agent and treatment by employing hyperthermia temperature. *J. Magn. Magn. Mater.* **564**, 170065 (2022).

[14] Shah, A. & Dobrovolskaia, M. A. Immunological effects of iron oxide nanoparticles and iron-based complex drug formulations: therapeutic benefits, toxicity, mechanistic insights, and translational considerations. *Nanomedicine: Nanatechnol. Biology Med.* **14**, 977–990 (2018). 10.1016/j.nano.2018.01.014 PMC5899012 29409836

[15] Wang, Y. X. J. Superparamagnetic iron oxide based MRI contrast agents: current status of clinical application. *Quant. Imaging Med. Surg.* **1**, 35–40 (2011). 23256052 10.3978/j.issn.2223-4292.2011.08.03 PMC3496483

[16] Wáng, Y. X. J. & Idée, J. M. A comprehensive literatures update of clinical researches of superparamagnetic resonance iron oxide nanoparticles for magnetic resonance imaging. *Quant. Imaging Med. Surg.* **7**, 88–122 (2017). 28275562 10.21037/qims.2017.02.09 PMC5337187

[17] Dadfar, S. M. et al. Iron oxide nanoparticles: diagnostic, therapeutic and theranostic applications. *Adv. Drug Deliv Rev.* **138**, 302–325 (2019). 30639256 10.1016/j.addr.2019.01.005 PMC7115878

[18] Auffan, M. et al. Towards a definition of inorganic nanoparticles from an environmental, health and safety perspective. *Nat. Nanotechnol*. **4**, 634–641 (2009). 19809453 10.1038/nnano.2009.242

[19] Laurent, S. et al. Magnetic iron oxide nanoparticles: synthesis, stabilization, vectorization, physicochemical characterizations, and biological applications. *Chem. Rev.* **108**, 2064–2110 (2008). 18543879 10.1021/cr068445e

[20] Dringen, R., Pawlowski, P. G. & Hirrlinger, J. Peroxide detoxification by brain cells. *J. Neurosci. Res.* **79**, 157–165 (2005). 15573410 10.1002/jnr.20280

[21] Maiorino, F. M. et al. Diversity of glutathione peroxidases. in 38–53 (1995). 10.1016/0076-6879(95)52007-4 10.1016/0076-6879(95)52007-4 7476373

[22] Aebi, H. Catalase in vitro. in 121–126 (1984). 10.1016/S0076-6879(84)05016-3 10.1016/s0076-6879(84)05016-3 6727660

[23] Latunde-Dada, G. O. & Ferroptosis Role of lipid peroxidation, iron and ferritinophagy. *Biochim. Biophys. Acta - Gen. Subj.* **1861**, 1893–1900 (2017). 28552631 10.1016/j.bbagen.2017.05.019

[24] Xie, W. et al. Shape-, size- and structure-controlled synthesis and biocompatibility of iron oxide nanoparticles for magnetic theranostics. *Theranostics* **8**, 3284 (2018). 29930730 10.7150/thno.25220 PMC6010979

[25] Leong, H. S. et al. On the issue of transparency and reproducibility in nanomedicine. *Nat. Nanotechnol*. **14**, 629–635 (2019). 31270452 10.1038/s41565-019-0496-9 PMC6939883

[26] Efremova, M. V. et al. Magnetite-Gold nanohybrids as ideal all-in-one platforms for theranostics. *Sci. Rep. 2018*. **81 8**, 1–19 (2018). 10.1038/s41598-018-29618-w PMC6062557 30050080

[27] N, H. et al. Correction: synthesis and preclinical application of a Prussian blue-based dual fluorescent and magnetic contrast agent (CA). *PLoS One* **18**, (2023). 10.1371/journal.pone.0295460 PMC10688837 38033129

[28] Zhang, X. Q. et al. Prussian blue modified iron oxide magnetic nanoparticles and their high peroxidase-like activity. *J. Mater. Chem.* **20**, 5110–5116 (2010).

[29] Jayalakshmi, M. & Scholz, F. Performance characteristics of zinc hexacyanoferrate/prussian blue and copper hexacyanoferrate/prussian blue solid state secondary cells. *J. Power Sources*. **91**, 217–223 (2000).

[30] Long, X. et al. Preparation, characterization and application in Cobalt ion adsorption using nanoparticle films of hybrid copper–nickel hexacyanoferrate. *RSC Adv.* **9**, 7485–7494 (2019). 35519994 10.1039/c9ra00596j PMC9061196

[31] Morozova, P. A. et al. Exploring the role of crystal water in potassium manganese hexacyanoferrate as a cathode material for potassium-ion batteries. *Crystals* **11**, 895 (2021).

[32] Salih, S. J. & Mahmood, W. M. Review on magnetic spinel ferrite (MFe_2_O_4_) nanoparticles: from synthesis to application. *Heliyon* **9**, e16601 (2023). 37274649 10.1016/j.heliyon.2023.e16601 PMC10238938

[33] Liandi, A. R. et al. Recent trends of spinel ferrites (MFe_2_O_4_: Mn, Co, Ni, Cu, Zn) applications as an environmentally friendly catalyst in multicomponent reactions: A review. *Case Stud. Chem. Environ. Eng.* **7**, 100303 (2023).

[34] Sonia, L. C. & Phanjoubam, S. Optical, magnetic and spin resonance studies of MFe_2_O_4_ (M = Mn, Co, Zn) ferrites. *Mater. Today Proc.* (2023). 10.1016/J.MATPR.2023.05.494

[35] Na, H., Bin, Song, I. C. & Hyeon, T. Inorganic nanoparticles for MRI contrast agents. *Adv. Mater.* **21**, 2133–2148 (2009).

[36] Sattarahmady, N., Heidari, M., Zare, T., Lotfi, M. & Heli, H. Zinc–Nickel ferrite nanoparticles as a contrast agent in magnetic resonance imaging. *Appl. Magn. Reson.* **47**, 925–935 (2016).

[37] Muhamad Arshad, J. et al. Synthesis and characterization of cobalt ferrites as MRI contrast agent. *Mater. Today Proc.* 47, S50–S54 (2021).

[38] Aoopngan, C. et al. Amine-Functionalized and Hydroxyl-Functionalized magnesium ferrite nanoparticles for congo red adsorption. *ACS Appl. Nano Mater.* **2**, 5329–5341 (2019).

[39] Ferrer, C. et al. Structural and magnetic studies of NiFe2O4 and NiFe_2_O_4_@SiO_2_-Silane agent samples useful for the removal of Cu^2+^ ions. *J. Alloys Compd.* **899**, 163403 (2022).

[40] Yadav, N. et al. Finite size effect on Sm^3+^ doped Mn_0.5_Zn_0.5_Sm_x_Fe_2–x_O_4_ (0 ≤ x ≤ 0.5) ferrite nanoparticles. *Ceram. Int.* **41**, 8623–8629 (2015).

[41] Ikramullah et al. Photocatalytic performance of zinc ferrite magnetic nanostructures for efficient eriochrome Black-T degradation from the aqueous environment under unfiltered sunlight. *Water Air Soil. Pollut*. **231**, 59 (2020).

[42] Yadav, R. S. et al. Sonochemical synthesis of Gd^3+^ doped CoFe_2_O_4_ spinel ferrite nanoparticles and its physical properties. *Ultrason. Sonochem*. **40**, 773–783 (2018). 28946484 10.1016/j.ultsonch.2017.08.024

[43] Cui, L. et al. Facile synthesis of Cobalt ferrite submicrospheres with tunable magnetic and electrocatalytic properties. *Colloids Surf. Physicochem Eng. Asp*. **423**, 170–177 (2013).

[44] Saleh, T. A., Majeed, S., Nayak, A. & Bhushan, B. Principles and advantages of microwave- assisted methods for the synthesis of nanomaterials for water purification. in *Advanced Nanomaterials for Water Engineering, Treatment, and Hydraulics* 40–57 (IGI Global, (2017). 10.4018/978-1-5225-2136-5.CH003

[45] Melo, R. S., Banerjee, P. & Franco, A. Hydrothermal synthesis of nickel doped Cobalt ferrite nanoparticles: optical and magnetic properties. *J. Mater. Sci. Mater. Electron.* **29**, 14657–14667 (2018).

[46] Azam, A. Microwave assisted synthesis and characterization of Co doped Cu ferrite nanoparticles. *J. Alloys Compd.* **540**, 145–153 (2012).

[47] Shukla, A. et al. Microwave assisted scalable synthesis of titanium ferrite nanomaterials. *J. Appl. Phys.* **123**, (2018).

[48] Wei, M., Huang, A. C., Shu, C. M. & Zhang, L. Thermal decomposition and nonisothermal kinetics of monoethanolamine mixed with various metal ions. *Sci. Rep.* **9**, (2019). 10.1038/s41598-018-38434-1 PMC6367447 30733558

[49] Chi, S. & Rochelle, G. T. Oxidative degradation of monoethanolamine. *Ind. Eng. Chem. Res.* **41**, 4178–4186 (2002).

[50] Li, Y. K., Cheng, L., Zhang, X. D. & Guo, X. Hierarchically-structured MnFe_2_O_4_ nanospheres for highly sensitive detection of NO2. *Solid State Ionics*. **336**, 102–109 (2019).

[51] Lee, Y. Fabrication of manganese ferrite (MnFe_2_O_4_) microsphere-coated magnetic Biochar composite for antimonate sequestration: characterization, adsorption behavior, and mechanistic Understanding. *Appl. Surf. Sci.* **578**, 152005 (2022).

[52] Flores-Lasluisa, J. X. et al. Electrocatalytic activity of calcined manganese ferrite solid nanospheres in the oxygen reduction reaction. *Environ. Res.* **204**, 112126 (2022). 34563521 10.1016/j.envres.2021.112126

[53] Nonkumwong, J., Ananta, S. & Srisombat, L. Effective removal of lead(II) from wastewater by amine-functionalized magnesium ferrite nanoparticles. *RSC Adv.* **6**, 47382–47393 (2016).

[54] Xu, Y., Sun, D., Hao, H., Gao, D. & Sun, Y. Non-stoichiometric Co(II), Ni(II), Zn(II)-ferrite nanospheres: size controllable synthesis, excellent gas-sensing and magnetic properties. *RSC Adv.* **6**, 98994–99002 (2016).

[55] Moreno-Castilla, C., López-Ramón, M. V., Fontecha-Cámara, M., ángeles, álvarez, M. A. & Mateus, L. Removal of Phenolic Compounds from Water Using Copper Ferrite Nanosphere Composites as Fenton Catalysts. *Nanomater.* Vol. 9, Page 901 9, 901 (2019). (2019). 10.3390/nano9060901 PMC6631195 31226850

[56] Gögen, Y. et al. Prussian blue modified magnetite decorated MW-CNT nanocomposite: synthesis, characterization and potential use as Photo-Fenton catalysts for phenol degradation. *Chem. Pap*. **78**, 1863–1874 (2024).

[57] Thammawong, C., Opaprakasit, P., Tangboriboonrat, P. & Sreearunothai, P. Prussian blue-coated magnetic nanoparticles for removal of cesium from contaminated environment. *J. Nanoparticle Res.* **15**, 1–10 (2013).

[58] Irving, H. & Williams, R. J. P. The stability of transition-metal complexes. *J. Chem. Soc.* 3192–3210. 10.1039/JR9530003192 (1953).

[59] Risset, O. N. et al. _j_M_k_[Fe(CN)_6_]_l_ (M = Co, Ni) Prussian blue analogue Hollow nanocubes: A new example of a multilevel pore system. *Chem. Mater.* **25**, 42–47 (2013).

[60] Divalent - an. overview | ScienceDirect Topics. https://www.sciencedirect.com/topics/pharmacology-toxicology-and-pharmaceutical-science/divalent

[61] Long, X. Y., Li, J. Y., Sheng, D. & Lian, H. Z. Spinel-type manganese ferrite (MnFe_2_O_4_) microspheres: A novel affinity probe for selective and fast enrichment of phosphopeptides. *Talanta* **166**, 36–45 (2017). 28213246 10.1016/j.talanta.2017.01.025

[62] Ilton, E. S., Post, J. E., Heaney, P. J., Ling, F. T. & Kerisit, S. N. XPS determination of Mn oxidation States in Mn (hydr)oxides. *Appl. Surf. Sci.* **366**, 475–485 (2016).

[63] Huang, X. et al. One-pot solvothermal synthesis of magnetically separable rGO/MnFe_2_O_4_ hybrids as efficient photocatalysts for degradation of MB under visible light. *Mater. Chem. Phys.* **231**, 68–74 (2019).

[64] Lv, Y., Ma, B., Liu, Y., Wang, C. & Chen, Y. A novel adsorbent potassium magnesium ferrocyanide for selective separation and extraction of rubidium and cesium from ultra-high salt solutions. *J. Water Process. Eng.* **55**, 104225 (2023).

[65] Mansour, A. N. et al. Structural analysis of K_4_Fe(CN)_6_·3H_2_O, K_3_Fe(CN)_6_ and Prussian blue. *ECS J. Solid State Sci. Technol.* **10**, 103002 (2021).

[66] Bohara, R. A., Thorat, N. D., Yadav, H. M. & Pawar, S. H. One-step synthesis of uniform and biocompatible amine functionalized Cobalt ferrite nanoparticles: a potential carrier for biomedical applications. *New. J. Chem.* **38**, 2979–2986 (2014).

[67] Gemeay, A. H., Keshta, B. E., El-Sharkawy, R. G. & Zaki, A. B. Chemical insight into the adsorption of reactive wool dyes onto amine-functionalized magnetite/silica core-shell from industrial wastewaters. *Environ. Sci. Pollut Res.* **27**, 32341–32358 (2020). 10.1007/s11356-019-06530-y 31707614

[68] Irfan, M. et al. Kinetics and adsorption isotherms of Amine-Functionalized magnesium ferrite produced using Sol-Gel method for treatment of heavy metals in wastewater. *Mater. 2022*. **15**, 4009 (2022). 10.3390/ma15114009 PMC9181868 35683307

[69] Lejeune, J., Brubach, J. B., Roy, P. & Bleuzen, A. Application of the infrared spectroscopy to the structural study of Prussian blue analogues. *Comptes Rendus Chim.* **17**, 534–540 (2014).

[70] Silva, W. O. et al. Oxidative print light synthesis thin film deposition of Prussian blue. *ACS Appl. Electron. Mater.* **2**, 927–935 (2020).

[71] Nawar, A. M. & Alzharani, A. A. Impedance spectroscopy and conduction mechanism analysis of bulk nanostructure Prussian blue pellets. *Mater. Chem. Phys.* **306**, 128000 (2023).

[72] He, X. et al. A novel highly crystalline Fe_4_(Fe(CN)_6_)_3_ concave cube anode material for Li-ion batteries with high capacity and long life. *J. Mater. Chem. A*. **7**, 11478–11486 (2019).

[73] Younes Jomma, E., Ding, S. N., Lei, Y., Tiwari, A. & Liu, H. One-Pot hydrothermal synthesis of magnetite Prussian blue Nano-Composites and their application to fabricate glucose biosensor. *Sens. 2016*. **16**, 243 (2016). 10.3390/s16020243 PMC4801619 26901204

[74] Tang, Z. X., Sorensen, C. M., Klabunde, K. J. & Hadjipanayis, G. C. Size-dependent curie temperature in nanoscale. *Phys. Rev. Lett.* **67**, 3602–3605 (1991). 10044777 10.1103/PhysRevLett.67.3602

[75] Gao, C. Y., Baek, E., You, C. Y. & Choi, H. J. Magnetic-stimuli rheological response of soft-magnetic manganese ferrite nanoparticle suspension. *Colloid Polym. Sci.* **299**, 865–872 (2021).

[76] Karaagac, O. & Köçkar, H. The effects of temperature and reaction time on the formation of manganese ferrite nanoparticles synthesized by hydrothermal method. *J. Mater. Sci. Mater. Electron.* **31**, 2567–2574 (2020).

[77] Praveena, K., Gowda, J. & El-Denglawey, G. V. Jagadeesha Angadi, V. Manganese ferrite—polyaniline nanocomposites for microwave absorbers in X band. *J. Mater. Sci. Mater. Electron.* **33**, 5678–5685 (2022).

[78] Aslibeiki, B., Kameli, P. & Ehsani, M. H. MnFe_2_O_4_ bulk, nanoparticles and film: A comparative study of structural and magnetic properties. *Ceram. Int.* **42**, 12789–12795 (2016).

[79] Tao, J. et al. Magnetic MnFe_2_O_4_/MoS_2_ nanocomposites synthesis for rapid degradation of sulfamethoxazole by activated peroxymonosulfate. *J. Taiwan. Inst. Chem. Eng.* **146**, 104777 (2023).

[80] Aslibeiki, B. & Kameli, P. Magnetic properties of MnFe_2_O_4_ nano-aggregates dispersed in paraffin wax. *J. Magn. Magn. Mater.* **385**, 308–312 (2015).

[81] Umut, E. Magnetic properties of manganese ferrite (MnFe_2_O_4_) nanoparticles synthesized by Co-Precipitation method. *Hittite J. Sci. Eng.* **6**, 243–249 (2019).

[82] Yang, L. X., Wang, F., Meng, Y. F., Tang, Q. H. & Liu, Z. Q. Fabrication and Characterization of Manganese Ferrite Nanospheres as a Magnetic Adsorbent of Chromium. *J. Nanomater.* 293464 (2013). (2013).

[83] Superparamagnetic Manganese Ferrite Nanoparticles. Synthesis and Magnetic Properties.

[84] Gerzsenyi, T. B. et al. A simplified and efficient method for production of manganese ferrite magnetic nanoparticles and their application in DNA isolation. *Int. J. Mol. Sci.* **24**, 2156 (2023). 36768483 10.3390/ijms24032156 PMC9917137

[85] Nitika, Rana, A. & Kumar, V. Influence of temperature on structural, magnetic and thermal properties of superparamagnetic MnFe_2_O_4_ nanoparticles. *Mater. Today Proc.* 45, 4773–4776 (2021).

[86] Ceylan, A. & Ozcan, S. Effects of disordered surface structure on the magnetic properties of nanocrystalline MnFe2O4. *Ceram. Int.* **41**, 3875–3878 (2015).

[87] Heydari, F. I. et al. Solvothermal synthesis of Polyvinyl pyrrolidone encapsulated, amine-functionalized copper ferrite and its use as a magnetic resonance imaging contrast agent. *PLoS One*. **20**, e0316221 (2025). 39913433 10.1371/journal.pone.0316221 PMC11801609

[88] Ilosvai, Á. M. et al. Development of Polymer-Encapsulated, Amine-Functionalized zinc ferrite nanoparticles as MRI contrast agents. *Int. J. Mol. Sci. 2023*. **24**, 16203 (2023). 10.3390/ijms242216203 PMC10671131 38003394

[89] Rohrer, M., Bauer, H., Mintorovitch, J., Requardt, M. & Weinmann, H. J. Comparison of magnetic properties of MRI contrast media solutions at different magnetic field strengths. *Invest. Radiol.* **40**, 715–724 (2005). 16230904 10.1097/01.rli.0000184756.66360.d3

[90] Chavhan, G. B., Babyn, P. S., Thomas, B., Shroff, M. M. & Haacke, M. Principles, techniques, and applications of T2*-based MR imaging and its special applications. *Radiographics* **29**, 1433–1449 (2009). 19755604 10.1148/rg.295095034 PMC2799958

[91] Punshon, G. et al. Interactions between endothelial cells and a poly(carbonate-silsesquioxane-bridge-urea)urethane. *Biomaterials* **26**, 6271–6279 (2005). 15913770 10.1016/j.biomaterials.2005.03.034

[92] Low, S. P., Williams, K. A., Canham, L. T. & Voelcker, N. H. Evaluation of mammalian cell adhesion on surface-modified porous silicon. *Biomaterials* **27**, 4538–4546 (2006). 16707158 10.1016/j.biomaterials.2006.04.015

[93] Shokouhimehr, M. et al. Dual purpose Prussian blue nanoparticles for cellular imaging and drug delivery: a new generation of T1-weighted MRI contrast and small molecule delivery agents. *J. Mater. Chem.* **20**, 5251–5259 (2010).

[94] Shokouhimehr, M. Prussian Blue Nanoparticles and its Analogues as New-Generation T1-Weighted MRI Contrast Agents for Cellular Imaging. (2010).

[95] Terry, L. & Riss, R. A. M. *Cytotoxicity assay*. (2006). https://patents.google.com/patent/US6982152B2/en?oq=Riss%2C+T.+L.%2C+%26+Moravec%2C+R.+A.+(2006). U.S.+Patent + No.+6%2C982%2C152.+Washington%2 C + DC:+U.S.+Patent + and + Trademark + Office.

